# Immune Cells in the BBB Disruption After Acute Ischemic Stroke: Targets for Immune Therapy?

**DOI:** 10.3389/fimmu.2021.678744

**Published:** 2021-06-23

**Authors:** Yan-mei Qiu, Chun-lin Zhang, An-qi Chen, Hai-ling Wang, Yi-fan Zhou, Ya-nan Li, Bo Hu

**Affiliations:** Department of Neurology, Union Hospital, Tongji Medical College, Huazhong University of Science and Technology, Wuhan, China

**Keywords:** immune cells, ischemic stroke, blood-brain barrier, inflammation, immune therapy

## Abstract

Blood-Brain Barrier (BBB) disruption is an important pathophysiological process of acute ischemic stroke (AIS), resulting in devastating malignant brain edema and hemorrhagic transformation. The rapid activation of immune cells plays a critical role in BBB disruption after ischemic stroke. Infiltrating blood-borne immune cells (neutrophils, monocytes, and T lymphocytes) increase BBB permeability, as they cause microvascular disorder and secrete inflammation-associated molecules. In contrast, they promote BBB repair and angiogenesis in the latter phase of ischemic stroke. The profound immunological effects of cerebral immune cells (microglia, astrocytes, and pericytes) on BBB disruption have been underestimated in ischemic stroke. Post-stroke microglia and astrocytes can adopt both an M1/A1 or M2/A2 phenotype, which influence BBB integrity differently. However, whether pericytes acquire microglia phenotype and exert immunological effects on the BBB remains controversial. Thus, better understanding the inflammatory mechanism underlying BBB disruption can lead to the identification of more promising biological targets to develop treatments that minimize the onset of life-threatening complications and to improve existing treatments in patients. However, early attempts to inhibit the infiltration of circulating immune cells into the brain by blocking adhesion molecules, that were successful in experimental stroke failed in clinical trials. Therefore, new immunoregulatory therapeutic strategies for acute ischemic stroke are desperately warranted. Herein, we highlight the role of circulating and cerebral immune cells in BBB disruption and the crosstalk between them following acute ischemic stroke. Using a robust theoretical background, we discuss potential and effective immunotherapeutic targets to regulate BBB permeability after acute ischemic stroke.

## Introduction

Stroke is the second leading cause of death worldwide and is characterized by a high rate of morbidity, mortality, and disability. Therefore, there is a substantial social and economic burden associated with stroke (1). Stroke is classified into two types: ischemic stroke and hemorrhagic stroke. Acute ischemic stroke (AIS) accounts for 87% of the total incidence of stroke, and is characterized by a sudden cessation of oxygen and blood supply due to arterial occlusion in local cerebral tissue (2). One of the hallmark pathophysiological features of ischemic stroke is blood-brain barrier (BBB) disruption, which is characterized by an increased permeability due to the degradation of tight junctions (TJs) and an enhancement in endothelial vesicle transport. As a result, there is an uncontrolled influx of blood-borne cells, macromolecules, and fluid, resulting in devastating cytotoxic and vasogenic edema and life-threatening hemorrhagic transformation (HT) (3–5). Ischemic stroke patients with severe BBB disruption present worse National Institutes of Health Stroke Scale (NIHSS) scores, functional prognosis, and increased mortality rates compared to those with mild BBB disruption (6, 7). As such, ameliorating BBB damage can improve neurologic outcomes in stroke patients (8).

In recent years, the understanding of the inflammatory mechanism of BBB disruption during AIS has improved. Ischemia manifests weakened inflammation-inhibiting signals, such as fractalkine [CX3C motif chemokine ligand 1 (CX3CL1)] as well as enhanced “help me” signals, such as damage-associated molecular patterns (DAMPs), from dying or necrotic neurons and glia, which may activate quiescent resident microglia, astrocytes, and pericytes by pattern recognition receptors (PRR) (9, 10). Activated cerebral immune cells upregulate the expression of pro-inflammatory factors and chemokines, as well as activate matrix metalloproteinases (MMPs) to compromise BBB integrity and recruit peripheral immune cells to the injured areas, leading to secondary BBB damage. In comparison, the transformation of immune cells to alternative phenotypes (N2 neutrophils, M2 macrophages, and A2 astrocytes) may protect the BBB against inflammatory injury by promoting the resolution of inflammation and angiogenesis, thereby re-establishing the integrity of the BBB in the latter phase of stroke. In addition, immune cells in the central nervous system (CNS) engage in crosstalk with infiltrated peripheral immune cells, forming a complicated inflammatory network that may indirectly influence BBB integrity. Therefore, circulating and cerebral immune cells play a profound and dual role in BBB disruption following ischemic stroke.

Currently, the first-line AIS therapies include intravenous alteplase(rt-PA) administration and mechanical thrombectomy (MT) (11). Medication-induced thrombolysis is the only approved thrombolytic therapy for AIS. However, MT is highly beneficial to AIS patients with large vessel occlusion. AIS reperfusion therapies by rt-PA and MT are limited to only 5 - 10% of patients because of the narrow window in which they are effective. There are only 4.5 hours after AIS onset for intravenous thrombolysis and up to 24 hours for mechanical thrombectomy, depending on the availability of imaging techniques to diagnosis AIS (4, 5). Because of the limited options for treatment of AIS, the identification of novel candidate drugs for treating AIS is an urgent challenge. Given the critical role of immune cells in BBB disruption, an in-depth exploration of the inflammatory mechanism of BBB disruption is crucial to identifying valuable therapeutic targets for treating ischemic stroke.

In this review, we first introduce the structure and function of the BBB. Then, we discuss the role of circulating and cerebral immune cells in BBB disruption and the crosstalk between them following acute ischemic stroke. Finally, we summarize the current immunoregulation targets to find potential and effective immunotherapy targets to regulate BBB destruction after acute ischemic stroke.

## Physiological Structure and Function of Blood-Brain Barrier

The BBB is a unique and tightly regulated anatomical interface between circulating blood and the CNS and is collectively formed by endothelial cells (ECs) the end-feet of astrocytes, and pericytes embedded in the basement membrane of capillary vessel (12). Continuous non-fenestrated ECs sealed by TJs constitute the innermost luminal side of the BBB (13). The ECs are surrounded by pericytes embedded in the capillaries and the end-feet of astrocyte that ensheathe the basement membrane. The intimate interactions between the ECs, pericytes, astrocytes, microglia, and neurons form the neurovascular unit, which is necessary for ensuring the functional integrity of the CNS (14).

The BBB plays a vital role in maintaining homeostasis in the neuronal microenvironment of the CNS through a number of different mechanisms. First, the BBB is a solid and highly regulated physical barrier that prevents exogenous neurotoxic components from entering the CNS. Distinguished from peripheral ECs, cerebral ECs form a continuous monolayer without fenestrations characterized by a low transcytosis rate and specified TJs (15, 16). TJs encompass three transmembrane proteins: claudins, occludin, and junction adhesion molecules (JAMs) (13, 17). Adherens junctions comprise transmembrane proteins, vascular endothelial (VE)-cadherin, with extracellular segments homotypically interacting and cytoplasmic domains binding to the plaque proteins, such as β-catenin, γ-catenin, and p120-catenin (18). Tight junctions (TJs) and adherens junctions form a circumferential zipper-like seal between adjacent ECs, positioning them as a gatekeeper for limiting paracellular permeability. The coverage ratio of pericytes in the brain is the highest among the vasculatures throughout the whole body (19). Due to the low permeability of the BBB, only small lipophilic molecules (<400kD) and gaseous molecules (e.g., N_2_, CO_2_, and O_2_) are capable of entering the cerebral parenchyma, while the delivery of blood-derived macromolecules into the cerebral parenchyma is severely limited (12).

The BBB is also an efficient and tightly regulated transport barrier that enables the delivery of essential nutrients to the CNS to meet the high-energy demands of neuronal activity. Cerebral ECs feature much higher numbers of mitochondria than peripheral ECs (12). Cerebral ECs also express thousands of ion channels, receptors, transporters, and active efflux pumps to selectively regulate molecular transport between the blood and the brain (20). In addition, the BBB is a molecular metabolic barrier. The unique vascular metabolism of the BBB ECs can change the solubility, reactivity, and transport properties of molecules. Last but not least, BBB is an immunologic barrier that blocks a variety of circulating leukocytes from entering the CNS and prevents CNS-specific antigens from infiltrating into the peripheral immune system. The extravasation of peripheral immune cells is dependent on adhesion molecules, such as vascular cell adhesion molecule-1 (VCAM-1) and intercellular cell adhesion molecule-1 (ICAM-1), which are expressed at extremely low levels in ECs and pericytes (21). Under normal physiological conditions, ECs suppress pro-inflammatory gene expression and quiesce circulating leukocytes (12). Therefore, the BBB has a direct role in regulating immune reactions within the CNS rather than act as a neutral and passive barrier. The BBB can modulate the function and fate of infiltrating immune cells in healthy states (22). The junctions of ECs, low expression region of the extracellular matrix(ECM), and gaps between pericytes are situated in parallel longitudinally at the venule of capillaries, forming a preferential pathway for enabling the diapedesis of peripheral leukocytes (23). Under systemic inflammatory conditions, such as in septic encephalopathy and neurodegenerative diseases, excessive immune responses damage TJs and ECs (24). Above all, the BBB acts as a physical barrier, transport barrier, metabolic barrier, and immunological barrier, all of which allow it to maintain delicate and narrow homeostasis in CNS.

## Role of Peripheral Immune Cells in the Disruption of BBB in Stroke

### The Temporal Trend of Peripheral Immune Cells Infiltrating the Brain

**Figure 1:**
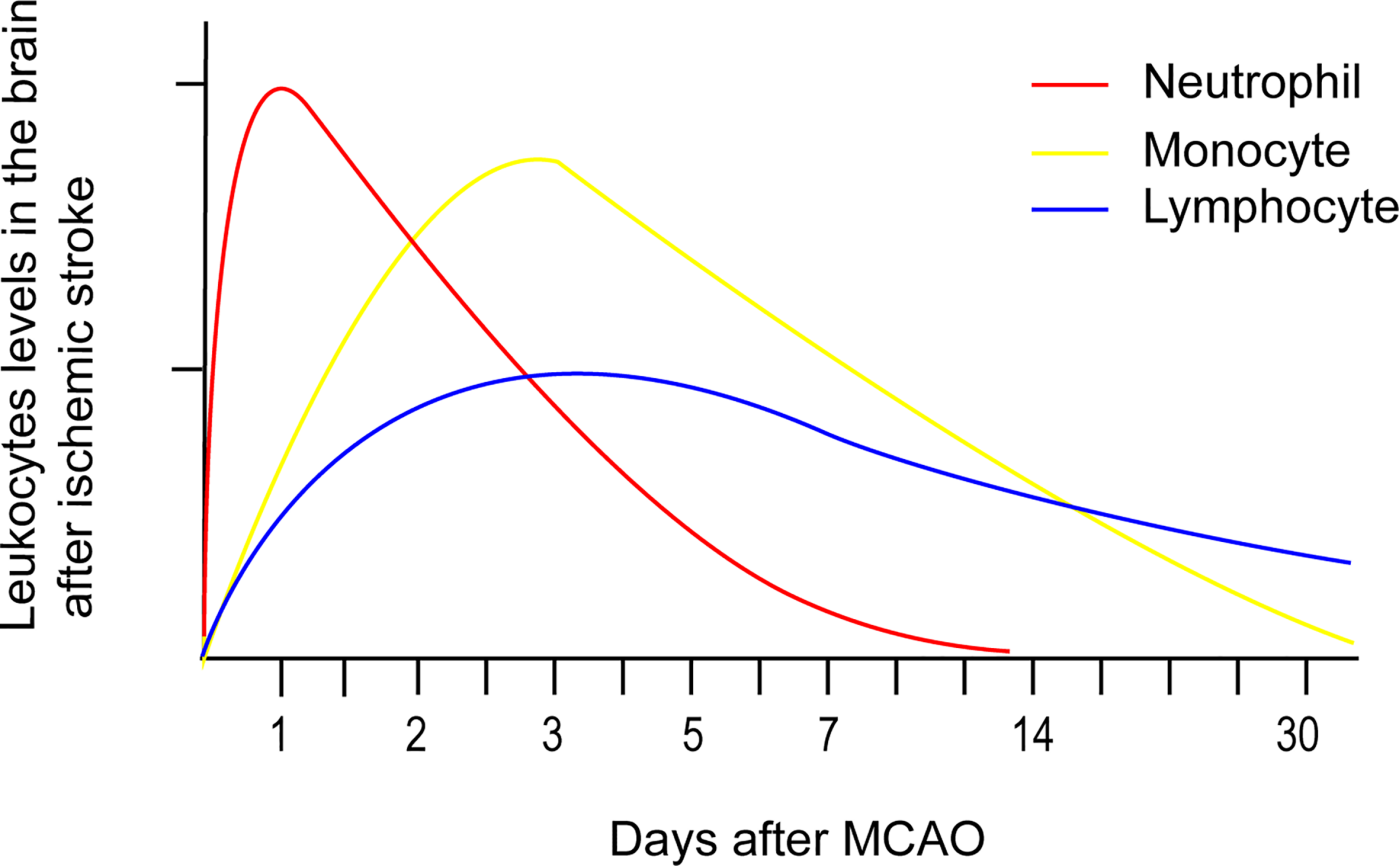
Temporal profile of peripheral immune cells accumulation after stroke onset based on experimental data. Neutrophil accumulation in the ischemic hemisphere increased significantly after 3 hours, reaching maximum accumulation after 24 hours, followed by a steady dissipation over 7 days. Monocyte counts in the ipsilateral hemisphere robustly increased after 1 day, peaked after 3 - 7 days, and then returned to baseline levels after 14 days. Lymphocytes extravasate into the injured hemisphere in smaller counts and longer persistence compared to the former two immune cells. The accumulation of T cells in the ischemic hemisphere significantly increased as early as 24 hours after AIS, peaked at 3 days, and persisted for 1 month.

Numerous peripheral immune cells infiltrate into the ischemic hemisphere in successive order after acute ischemic stroke. Circulating neutrophil counts displayed an exponential increase within a few hours after symptom onset and remained elevated for one week (25). Experimental studies have shown that neutrophil accumulation in the ischemic hemisphere increased significantly after 3 hours, reaching maximum accumulation after 24 hours, followed by a steady dissipation over 7 days (**Figure 1**) (26). The levels of strong neutrophil chemoattractants, such as chemokine-like factor 1 (CKLF-1), C-X-C motif chemokine ligand 1 (CXCL1), C-X-C motif chemokine ligand 2 (CXCL2), C-X-C motif chemokine ligand 5 (CXCL5), monocyte chemoattractant protein-1/C-C chemokine ligand 2 (MCP-1/CCL2), C-C chemokine ligand 3(CCL3), and C-C chemokine ligand 5 (CCL5), all increased dramatically after stroke. These chemoattractants enable neutrophils to be the first blood-borne immune cells to migrate to the injured brain tissue by binding to C-C chemokine receptor 5 (CCR5) and C-X-C chemokine receptor 1 (CXCR1) on the surface of neutrophils (27). CKLF-1 increased significantly at 3 hours poststroke, which bind to CCR5 on the surface of neutrophils. The binding of CKLF-1 to CCR5 mediate neutrophils migration through the Akt/GSK-3β and mitogen-activated protein kinase (MAPK) pathways (28).

Overall monocyte counts were dramatically increased in the blood of patients with acute ischemic stroke over the course of 16 days after onset. In addition, monocyte counts (**Figure 1**) in the ipsilateral hemisphere robustly increased after 1 day, peaked after 3 - 7 days, and then returned to baseline levels after 14 days (29). Monocyte recruitment is greatly dependent on CCR2, the receptor on surface of the classical monocytes that undergoes ligation with its ligand CCL2. T lymphocyte counts in the blood of acute ischemic stroke patients exponentially declined over 7 days, with the lowest count observed after 12 hours (25). Lymphocytes extravasate into the injured hemisphere in smaller counts and longer persistence compared to the former two immune cells. The accumulation of T cells (**Figure 1**) in the ischemic hemisphere significantly increased as early as 24 hours after AIS, peaked at 3 days, and persisted for 1 month (30). Double negative T cell (DNT) and CD8^+^T cell infiltration increased significantly for 3 - 24 hours following ischemic insult (26), while infiltrating CD4+T cells did not increase until 24 hours in a permanent middle cerebral artery occlusion (pMCAO) model (31). Regulatory CD4^+^ T cells (Tregs) were found to increase in the lesion area in a delayed manner after several days and persisted 30 days after stroke (32). Natural killer (NK) cells infiltrated the ischemic region as early as 3 hours after stroke, peaked at 12 hours, and remained elevated for at least 4 days (33). The attraction of Treg and gammadeltaT cells (γδT cell) were mediated by the CCL5/CCR5 and CCL6/CCR6 axes separately (34, 35). NK cells were recruited to the ischemic region through the IP-10/CXCR3 and CX3CL1/CX3CR1 axes (36).

### Intravascular Immune Cells Exert Deleterious Effects on BBB Disruption After Acute Ischemic Stroke

Neutrophils roll and adhere to ECs at venules to occlude blood flow, a process known as no-reflow phenomenon. Myeloid-specific α9-deficiency, which is a method to uniquely block neutrophil adhesion, remarkedly decreased the infarct volume and neurological deficit in a murine transient middle cerebral artery occlusion (tMCAO) model (37). Polymorphonuclear cells were detected in capillaries and post-capillary venules in the ipsilateral hemisphere as early as 30 minutes after stroke and reached a peak at 12 hours (38). P-selectin glycoprotein ligand-1 (PSGL-1) and macrophage-1 antigen (Mac-1) were elevated on the surface of neutrophils within a few hours after ischemic stroke (39). Meanwhile, P-selectin and ICAM-1 were up-regulated on ECs. PSGL-1/P-selectin and Mac-1/ICAM-1 interactions between neutrophils and ECs not only caused no-reflow but also increased paracellular permeability (40, 41). Upon adhesion, ICAM-1 induced a series of dedicated downstream pathways within ECs, including transient increases in the concentration of intracellular free calcium, subsequent myosin light chain kinase (MLCK) activation, focal adhesion kinase phosphorylation, and small GTPase Rho/ROCK activation, further enhancing actin polymerization and the breakdown of adherens junctions (42). Thus, neutrophil adhesion resulted in the contraction of ECs and paracellular hyperpermeability. In addition, neutrophil-platelet aggregations mediated by transcellular interactions between Mac-1and the GPIb counter receptor also increased thrombus formation and BBB permeability in experimental stroke (43).

T cells infiltrated the human brain profoundly for as long as 14 days following stroke (30). Upon arriving at the microvascular interface of artery occlusion, T cell capture, roll, arrest, crawl, and across the BBB through interactions with ECs *via* PSGL/P-selectin, very late antigen 4 (a4β1)/VCAM-1, function-associated antigen-1 (LFA-1)/ICAM-1 signaling (44). Receptor responsible for recruiting and extravasating including A kinase anchoring protein 7 (AKAP7) and CD74, were upregulated in T cells in patients with AIS. The increased expression of AKAP7 and CD74 was associated with enhanced hyperintense acute reperfusion marker (HARM), infarction size, and NIHSS score (45, 46). HARM is a delayed enhancement of the cerebrospinal fluid space that is observed on post-contrast MRI and is indicative of BBB disruption. Intravascular T lymphocytes interplay with ECs and platelets to facilitate the adhesion of platelets and leukocytes leading to microthrombus formation, which is known as thrombo-inflammation. Intravascular Tregs were found to induce enhanced microvascular dysfunction *via* the LFA-1/ICAM-1 pathway, which increased the infarction damage within 24 hours in a tMCAO model (47). When penetrating the cerebral parenchyma, T lymphocytes produced MMPs, reactive oxygen species (ROS), and pro-inflammatory factors to degrade the extracellular matrix (ECM) and damage ECs (48). However, T lymphocytes may also protect BBB integrity and prevent HT by binding to P-selectin on the surface of platelets (49).

### Neutrophil

#### Neutrophils and BBB Disruption in Human AIS

Neutrophils are well-known for their destructive role in BBB disruption after ischemic stroke. Clinical observations support the detrimental effects of neutrophils on the integrity of the BBB after ischemic stroke. The elevated neutrophil-to-lymphocyte ratio (NLR) in circulation at admission is an essential predictor of HT, especially parenchymal hematoma and symptomatic intracranial hemorrhage (sICH) (50, 51). Higher NLR was independently correlated to early neurological deterioration 24 hours post-stroke, as well as poor functional outcome and mortality after 3 months in patients with AIS (52, 53). Higher neutrophil counts and myeloperoxidase (MPO) plasma concentration in plasma were positively correlated with stroke severity and worsened outcomes (54).

Evaluating the function of circulating neutrophils in patients with AIS can reveal the overwhelming intracellular change in neutrophils in AIS. The percentage of the overactive senescent neutrophil subpopulation increased following ischemic stroke, which was characterized by elevated adhesion molecule and elastase expression as well as ROS production (55). Transcriptomic analysis revealed that mammalian target of rapamycin (mTOR), oxidative phosphorylation, growth factor signaling, and calpain proteases signaling pathways were generally up-regulated in circulating neutrophils of ischemic stroke patients (56). MMP-9-positive neutrophils were detected extensively surrounding the cerebral microvessels in the hemorrhagic and infarcted regions of patients with fatal HT after ischemic stroke, which was associated with high MMP9 expression levels and severe basal lamina collagen IV degradation (57). Neutrophil extracellular traps (NETs), whose components are mainly derived from neutrophils, were significantly increased in the plasma of ischemic stroke patients, associated with stroke severity and mortality (58). By immunostaining analysis, NET constituted more than 13% of the whole thrombi from patients subjected to endovascular thrombectomy (59).

#### Role of Neutrophils in BBB Disruption in AIS: Preclinical Evidence

Targeting neutrophils in rodents subjected to experimental stroke significantly decreased the infarct volume. Myeloid Mcl1 ablation protected mice from ischemic injury by selectively inhibiting the presence and infiltration of neutrophils (60). Doxorubicin (DOX)-conjugated protein nanoparticles (NPs) selectively targeted inflammatory neutrophils to induce apoptosis. Administration of DOX-NPs notably restored behavioral outcome in a cerebral ischemic/reperfusion model (61). These clinical and experimental stroke results suggested that, not only were there strong links between neutrophils and BBB disruption, but also targeting neutrophils may protect the BBB against inflammatory injury to improve stroke outcomes.

**Figure 2:**
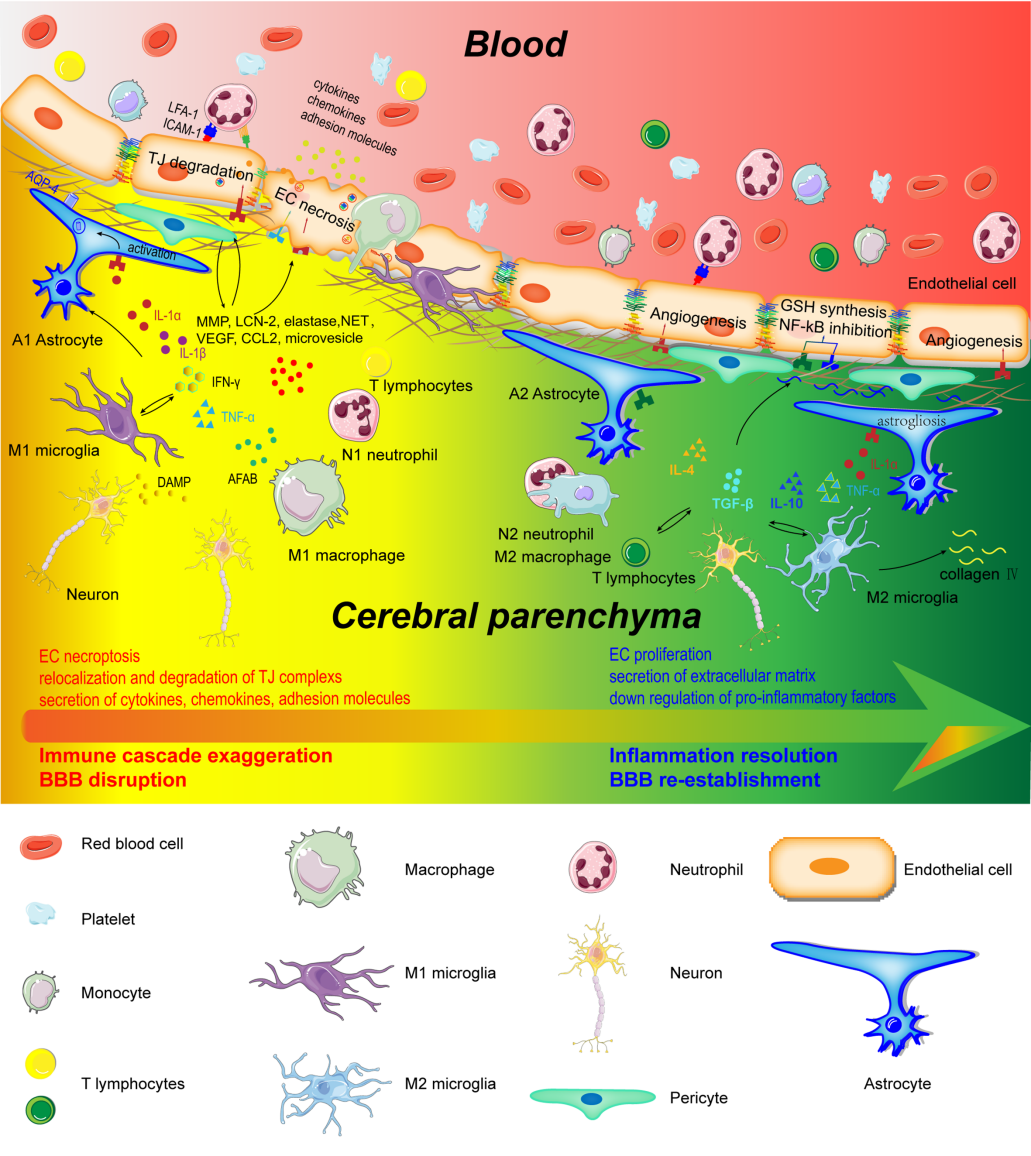
Schematic representation of cerebral and peripheral immune cells regulating BBB integrity during early and later phases of ischemic stroke. Infiltrated neutrophils produce proteases (MMPs, proteinase 3, and elastase), lipocalin-2, NET, microvesicles, cytokines, and chemokines to destroy the BBB structure. M1-type monocytes secret cytokines and chemokines to degrade TJs. Perivascular microglia phagocyte ECs, which directly lead to endothelial dysfunction and BBB disintegration. In addition, M1 microglia disrupt BBB integrity through the production and secretion of pro-inflammatory factors (IL-1α, IL-1β, IL-6, TNF-α, IFN-γ, and CCL2), MMP9, and VEGF. A1 astrocytes directly exert deleterious effects on BBB through increasing VEGF, cytokines (IL-1β, IL-6, and TNF-α), chemokines (CCL2 and CCL5), MMP, and LCN-2. However, in the recovery phase of AIS, these immune cells contribute to inflammation resolution and BBB re-establishment. N2 neutrophils promote engulfment of neutrophils by macrophages and inflammation resolution. Monocyte-derived M2 macrophages facilitate the expression of collagen IV and efferocytosis. Microglia directly protect BBB integrity through the secretion of IL-10 and TGF-β. A2 astrocytes are capable of secreting IL-2, IL-10, and TGF-β to accelerate inflammation resolution.

Neutrophils potentiate BBB disruption in many ways (**Figure 2**). First, it is well-known that neutrophils produce ROS, proteases (MMPs, proteinase 3, elastase), lipocalin-2(LCN-2), and NETs to degrade the BBB structure. Excessive ROS (e.g., superoxide anions, peroxynitrite, and hydrogen peroxide) production engenders damages to junction proteins (mainly cadherin-β-catenin complex, occludin, ZO-1, and claudin-5) and a reorganization of the endothelial cytoskeleton through hyperpermeability associated signaling pathways (e.g., MLCK, PKC, MAPK, and Rho GTPases) (62). Proteases, including MMPs, proteinase 3, and elastase were also released during neutrophil degranulation during ischemic stroke. MMPs directly facilitate the degradation of the BBB structure by cleaving glycocalyx, VE-cadherin, focal adhesion components, and basement membrane proteins, such as collagen IV (63). LCN-2, also termed neutrophil gelatinase-associated lipocalin, was detected in the serum of tMCAO mice as early as 1 hour following occlusion. The level of LCN-2 peaked after 24 hours and then subsided after 48 hours in the ipsilateral hemispheres (64). LCN-2 exacerbateed Evans blue (EB) extravasation and brain edema by reducing the expression of junction proteins, namely claudin-5 and β-catenin, during AIS. Therefore, LCN-2 neutralization can attenuate BBB disruption (65). However, another study found that LCN-1 reversed the decreased endothelial translocation of ZO-1 and VE-cadherin from the membrane to the cytoplasm of ECs after tumor necrosis factor-alpha (TNF-α) treatment, which mitigated BBB disruption (66). This suggested that LCN influences BBB permeability through various mechanisms under different situations.

NETs are web-like structure comprising nuclear and granular contents, mainly double-stranded DNA and granule proteins, such as MPO and elastase. H3Cit is a common biomarker of NET formation. The concentration of H3Cit began to increase as early as 6 hours after AIS onset and was present its highest concentration after 3 - 5 days, with distribution predominantly in the peri-infarct cortex. In fact, 78.7% of H3Cit was derived from neutrophils. NETs increased BBB permeability and extravascular IgG deposits, and decreased pericyte coverage in experimental stroke (67).

Second, neutrophils play a detrimental role in BBB integrity during AIS through the production of neutrophils-derived cytokines [e.g., interleukin-1β (IL-1β), interleukin-6 (IL-6), interleukin-8 (IL-8), and TNF-α) and chemokines (CCL2, CCL3, and CCL5)] (68). CCL2, also known as MCP-1, reduced the transendothelial electrical resistance and EB extravasation through TJ disassembly *via* the internalization of occludin and claudin-5 in the p-p38 MAPK signaling pathway (69, 70).

Furthermore, neutrophil-derived microvesicles were found to alter the transcriptomic profile of human cerebral microvascular ECs to dysregulate TJ and versicle transport *in vitro* (71).

Interestingly, recent evidence has suggested that neutrophils may adopt an anti-inflammatory phenotype by expressing of Ym1 and CD206 (72). Accelerated by peroxisome proliferator-activated receptor gamma (PPAR-γ) agonists while being hampered by Toll-like-receptor (TLR) activation, the N2 polarization of neutrophils promoted the engulfment of neutrophils by microglia/macrophages, which led to reductions in brain edema and infarct volume (73, 74). Neutrophils may also have a dual function in the evolution of cerebral ischemic damage by accelerating inflammatory response or the resolution of inflammation. Skewing neutrophils toward the N2 phenotype rather than comprehensively suppressing the infiltration and function of neutrophils may be a novel and promising strategy for treating ischemic stroke in the future.

### Monocytes

#### Monocytes and BBB Disruption in Human AIS

The exact role of monocytes in BBB disruption remains unclear, since clinical observations are controversial. Some studies suggested that their roles were harmful to BBB disruption. Because increased numbers of circulating monocytes were associated with HARM, larger AIS volume, worsened NIHSS score, and poorer outcomes compared to lower numbers of circulating monocytes (75). Higher monocyte-to-HDL (high-density lipoprotein) ratios (MHR) and monocyte-to-lymphocyte ratios (MLR) were associated with higher HT risk and poorer outcomes compared to lower ratios (76, 77). Monocytes were found to be accumulated and randomly distributed in erythrocytic thrombi, engendering a higher NIHSS score (78). However, other investigations argued for the opposite view. Because monocyte counts did not change during AIS, the counts cannot predict long-term mortality (79, 80). A recent investigation demonstrated that lower MHRs were independently associated with increased HT risk, especially sICH in AIS patients (81). The possible explanation for these paradoxical results may be the functional heterogeneity. In human, there are three monocyte subsets: classical CD14^++^CD16^−^, intermediate CD14^+^CD16^+^, and alternative/non-classical CD14^+^CD16^++^. However, in mice, there are two subpopulations: classical Ly6C^+^CCR2^high^CX3CR1^low^ and alternative/non-classic Ly6C^−^CCR2^low^CX3CR1^high^. Elevated levels of classical monocytes were associated with early clinical worsening and higher mortality in ischemic stroke, while non-classical monocytes were significantly decreased in stroke patients. Decreased levels of non-classical monocytes was inversely related to poor prognosis (82). Thus, distinct subpopulations may exert different roles in BBB integrity and stroke outcome.

#### Role of Monocytes in BBB Disruption in AIS: Preclinical Evidence

In experimental stroke, inhibiting monocyte recruitment by blocking or depleting CCR2 significantly protected against brain edema but impaired long-term recovery (83, 84). Pro-inflammatory monocytes were the predominant monocytes subsets infiltrating into the injured brain, while alternative monocytes tend to be redundant in AIS (85, 86). However, it remains unclear what role non-classical monocytes play in BBB disruption after AIS.

This counterintuitive result might be explained by the phenotype transformation of monocytes. The phenotype and function of blood-derived monocytes were not univariable during AIS, depending on the inflammatory context. The phenotype of monocyte shifted from pro-inflammatory M1 dominant day 3 to anti-inflammatory M2 dominant 7 days after stroke, indicating a functional transformation of the monocytes from amplifying the immune response to the resolution of inflammation (87). Infiltrating monocytes subsequently down-regulated Ly6C, up-regulated F4/80, and acquired macrophage features. Interestingly, M2 macrophages can be further subdivided into M2a, M2b, and M2c (88, 89), and some authors also classify the M2d subtype (90). M2a macrophages were typically induced by IL-4 and IL-13. IL-13 administration effectively promoted the transformation of macrophages from the M1 state to the M2a state at 3 days following permanent ischemia in mice (91). M2b macrophages were induced by the immunoglobulin Fcγ receptor, lipopolysaccharide and IL-1β complex. M2c macrophages were induced by the anti-inflammatory cytokine IL-10 and glucocorticoids, while M2d macrophages were induced by the stimulation of IL-6 and adenosine.

Monocytes destroy BBB integrity in the early phase of AIS (**Figure 2**). Monocytes were first observed within the injured region of capillaries and venules 4-6 hours after occlusion in rats (38). M1-type monocytes secrete ROS, cytokines, and chemokines through the activation of the inflammasome to degrade TJs between ECs in the BBB. The polarization of the M1 pro-inflammatory phenotype was promoted through the activation of acute purinergic receptor P2X4 (P2X4R) (92). Blood-borne monocytes exacerbated secondary inflammatory damage to the BBB by up-regulating triggering receptor expressed on myeloid cells 1, assisting other innate immune receptors (93). In addition, monocytes upregulated adipocyte fatty acid-binding protein expression to potentiate the MMP9-mediated degradation of TJs by enhancing JNK/c-Jun signaling (94). Thus, monocytes can damage BBB in a paracrine manner.

On the other hand, monocyte-derived M2 macrophages may protect the BBB against ischemic damage by vascular remodeling, physical attachment, and inflammation resolution. RNA sequencing revealed the overactivation of JAK1, JAK3, and STAT3, as well as angiogenesis in monocytes (56, 95). Monocytes and macrophages with elongated morphologies were observed lining the vessels in the infarct core and peri-infarction regions (85). The adhesion of macrophages to ECs directly pulled the ruptured ends, narrowing the lesion through the polymerization of microfilaments and phosphatidylinositide 3-kinase- or Rac1- mediated mechanical traction forces (96). All four M2 macrophages subtypes acquired enhanced phagocytic ability and expressed IL-10, contributing to the resolution of inflammation. Once differentiated into mature phagocytes, monocytes were found to promote the expression of collagen IV to prevent HT in the smad2/TGF-β signaling pathway (97). Furthermore, efferocytosis-related genes were robustly up-regulated in macrophages (98). Therefore, hematogenous macrophages contribute to BBB recovery indirectly through inflammation resolution and efferocytosis.

Despite being an M2 subtype, the function of macrophages may be diverse. M2a macrophages express various anti-inflammatory and neurotrophic factors such as arginase 1 (Arg1) and insulin-like growth factor-1. M2c macrophages increase the expression of TGF-β, CD163, and sphingosine kinase. IL-13 induced transition from M1 macrophage to M2a macrophages attenuated the pro-inflammatory cascade and neurological disorders (91). However, M2b macrophages increase the production of pro-inflammatory factors including IL-1β, IL-6, TNF-a, which may potentiate inflammation and increase the BBB permeability in the early phase of AIS (99). M2d macrophages secrete VEGF-A and TNF-α, all of which were found to be deleterious to BBB integrity in AIS. Investigations on the specific roles of these different M2 macrophages in BBB disruption during AIS are relatively lacking. Therefore, more in-depth studies are warranted to develop more insights into these processes.

### T Lymphocytes

#### The Gut-Brain Axis Contributes to the Peripheral T Lymphocytes Subpopulation Disorder and BBB Disruption

Clinical investigations revealed that the differentiation of T lymphocyte pools skewed to pro-inflammatory subpopulations after AIS. T lymphocytes represent a conglomeration of different subpopulations with tremendous heterogeneity. The percentage of immunosuppressive Tregs was significantly decreased in response to AIS, which was associated with poor stroke outcome (100). CD39^+^ Tregs are typically representative of functionally active Tregs and are the most strongly reduced subpopulation of T lymphocytes after stroke, indicating that the immunosuppressive function of Tregs was largely impaired during AIS (101). Thus, Tregs-derived anti-inflammatory factors, including transforming growth factor-beta (TGF-β) and interleukin-10(IL-10), were reduced after stroke (102). In contrast, the increased proportion of pro-inflammatory T helper 17 (Th17) cells and γδT cells in the serum of AIS patients was accompanied by elevated levels of pro-inflammatory factors, such as interleukin-17A (IL-17A), interleukin-23 (IL-23), IL-6, and IL-1β (102, 103). CD4^+^CD28^-^ T cells have the potential to amplify immune responses and tissue damage. The significantly increase in CD4^+^CD28^-^ T cells was associated with a higher recurrence/death rate in ischemic stroke (104). The levels of Tim-3, a specific marker of T-helper 1 (Th1) cells, were elevated, along with TNF-α and IL-17, in circulating blood (105). These Th1 cells may participate in the pathogenesis of AIS by enhancing the immune cascade.

Interestingly, the gut-brain axis, which represents the interaction between the brain and the gut, was recently shown to demonstrate a causal relationship between abnormalities in T cell subpopulation and BBB disruption during ischemic stroke. Acute infarction in the brain induced microbiota dysbiosis in the gut. Patients with severe ischemic stroke showed a more significantly reduced intestinal diversity compared to those without AIS, with the enrichment of trimethylamine-*N*-oxide (TMAO)-producing bacteria, opportunistic pathogens, and loss of bacteria implicated in butyrate production (106, 107). In turn, gut microbiota dysbiosis resulted in deteriorated stroke outcomes. The plasma TMAO levels of AIS patients increased significantly post-stroke, and these elevated levels were associated with a worse modified Rankin Scale (mRS) score after 3 months (108). However, other studies demonstrated that TMAO levels in blood declined or did not change in the plasma of AIS patients (109). TMAO intensified ischemic damage through three main mechanisms: by increasing the proportion of proinflammatory monocytes; by activating the nucleotide-binding oligomerization domain-like receptor family pyrin domain-containing 3 (NLRP3) inflammasome to amplify the immune response, and by stimulating ROS production in the mitochondria to damage ECs (110, 111).

The mechanism underlying the attenuation of peripheral T lymphocytes and BBB disruption was explored in experimental stroke. More than 70% of immune cells were pooled in the gut, where the balance of the peripheral T lymphocytes was skewed toward pro-inflammatory cells after ischemic stroke, from where the lymphocytes migrate to the cerebral infarction area. The histamine (HA)/gut histamine receptor mediated the elevation of pro-inflammatory factors [IL-6, TNF-α, Interferon-gamma (IFN-γ), and granulocyte-colony stimulating factor (G-CSF)] in the gut, facilitating the differentiation into pro-inflammatory T cells (112). The gut microbiota dysbiosis also abolished the ability of Dendritic cells (DCs) to drive Treg differentiation and promoted the ability of DCs to induce γδT cell differentiation (113). Therefore, targeting the gut microbiota may be a promising treatment for ischemic stroke.

#### The Disturbed Balance Between Pro-Inflammatory Subsets and Anti-Inflammatory Subsets Contributes to BBB Disruption: Preclinical Evidence

**Figure 3:**
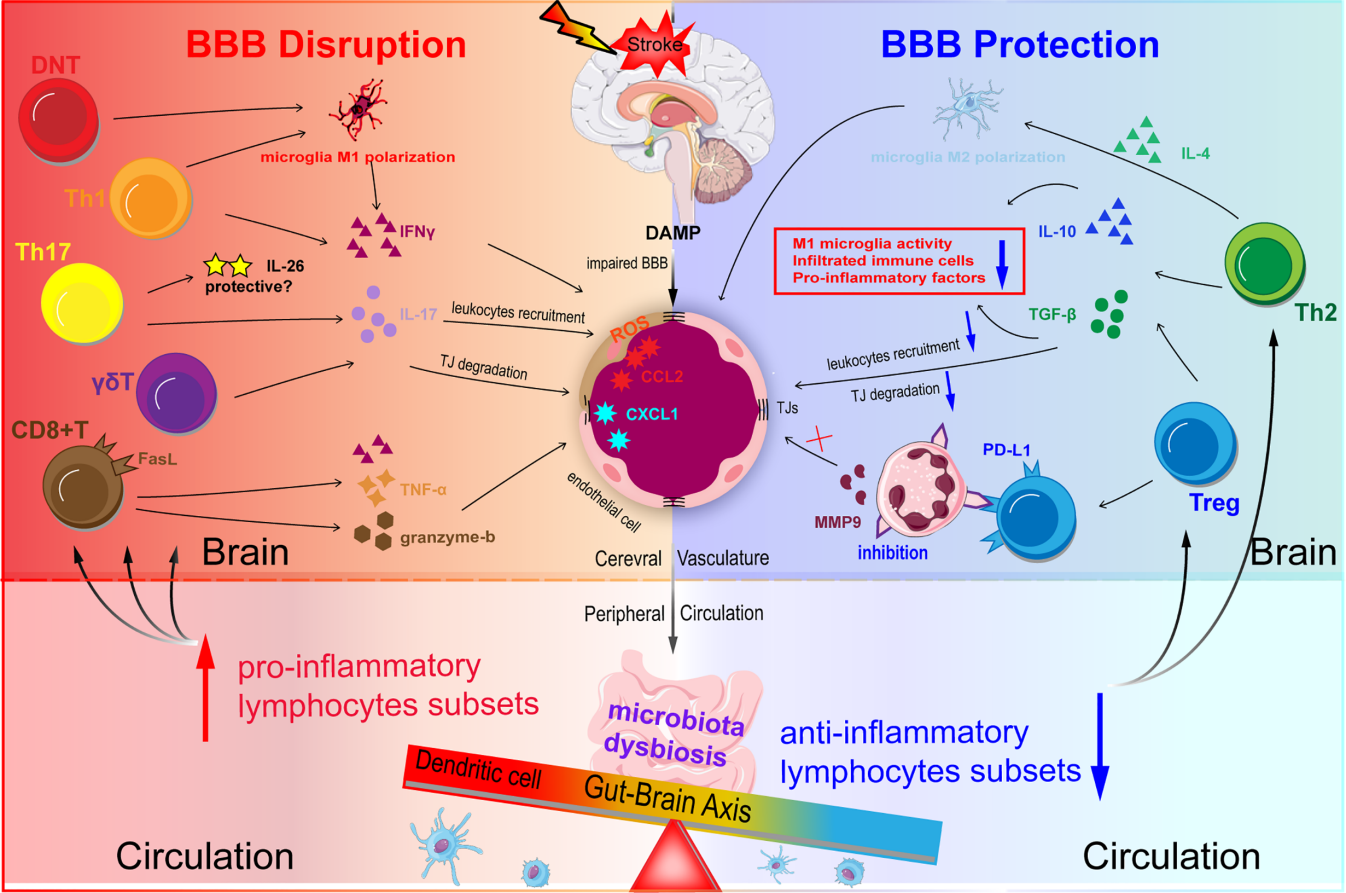
Schematic representation of T lymphocytes homeostasis disorder regulating BBB integrity in ischemic stroke. DAMP and chemokines are released from the ischemic brain *via* impaired BBB. Acute infarction in the brain can induce microbiota dysbiosis in the gut. Microbiota dysbiosis abolishes the ability of Dendritic cells (DC) to drive the Treg differentiation and promotes the power of DC to induce γδT cell differentiation. The disturbed balance between pro-inflammatory subsets and anti-inflammatory subsets contributes to BBB disruption during ischemic stroke. Th1 and Th17 may degrade TJs and destroy BBB integrity through secreting IFN-γ, IL-17, and IL-21 in the acute phase. CD8^+^T cells potentiate the ischemic stroke progression by two main methods: one is granzyme-b and FasL induced cytotoxicity through FasL-PDPK1 pathway and the other is TNF-α and IFN-γ. Treg suppresses the overactivation of resident microglia, infiltrated T cells, and neutrophils, and decrease pro-inflammatory factors (TNF-α, IFN-γ, IL-1β) levels mainly through the action of IL-10 and TGF-β.

The enhanced and elevated pro-inflammatory T cells accompanying AIS directly degrade the BBB through the production of cytokines (**Figure 3**). Th1 and Th17 cells degraded TJs and compromised BBB integrity by secreting IFN-γ, IL-17, and IL-21 in the acute phase of ischemic stroke (114, 115). γδ T cells have been reported to be pathogenic in stroke outcomes through IL-17. IL-17 deteriorated BBB integrity by decreasing the expression of occludin and ZO-1, and it facilitated the recruitment of monocytes and neutrophils by increasing CCL2 and CXCL1 expression in ECs (116). IL-17 also reduced the expression of TJs in ECs by increasing ROS production in an NADPH oxidase- or xanthine oxidase-dependent manner (117). Interestingly, Th17 cells produced IL-26, a BBB-protective cytokine, that up-regulated the expression of occludin, claudins, and ZO-1 in ECs in an *in vitro* BBB model (118). However, whether or not IL-17 protects intact BBB in ischemic stroke remains unknown. NK cells significantly increased cerebral microvascular EC permeability under oxygen-glucose deprivation (OGD) conditions (33). NK cells aggregated the ischemic lesion through the production of IFN-γ and ROS (36). Previous studies confirmed that CD8^+^T cells potentiated the progression of ischemic stroke by granzyme-b and FasL induced cytotoxicity and by TNF-α and IFN-γ (119). Recently, Fudong Shi’s lab reported that CD8^+^T cells engendered an increased EC permeability by degrading claudin-5 as early as 1 hour after OGD treatment (120). However, the exact and comprehensive effects of CD8+T cells on BBB integrity remain much to be explored.

In contrast to pro-inflammatory T cells, the immunosuppressive functions of anti-inflammatory T cells were impaired in ischemic stroke. Tregs and Th2 shield the ischemic hemisphere from causing an overactive immune response and aggravating secondary injury progression after AIS. Tregs suppressed the overactivation of resident microglia and infiltrated T cells and decreased the levels of pro-inflammatory factors (TNF-α, IFN-γ, IL-1β) mainly *via* upregulated expression of IL-10 and TGF-β (121). Interestingly, Tregs directly inhibited the production of MMP9 in neutrophils by transcellularly binding to neutrophils *via* the program death 1-ligand 1 (PD-L1), which resulted in an improved BBB integrity and neurological performance (122). Tregs also significantly attenuated BBB damage and functional deficits by downregulating the expression of endothelial CCL2 and MMP9 (123).

## Role of Cerebral Immune Cells in the BBB Disruption in AIS: Preclinical Evidence

Microglia form the largest immune cell population in the brain and feature small cell soma that are highly ramified, enabling them to continually scan the surrounding environment (124). The activation of microglia was restrained by neurons *via* CX3CL1/CX3CR1 signaling pathway in healthy brains (125). Astrocytes are another type of cerebral immune cells and express significant amounts of PRRs, and TLRs, as well as mannose, scavenrer, and NOD-like receptors. Astrocytes also secrete cytokines, chemokines, adhesion molecules, and proteases to regulate BBB integrity and form glial scars in response to danger signals (126). In contrast, the role of pericytes in BBB disruption has been investigated from a different perspective recently. Pericytes consistently express PRRs on their surface to induce IL-1β secretion when activated by danger signals and pro-inflammatory factors (127). Pericytes also regulate the diapedesis and migration of leukocytes at the post-capillary venule of the BBB and engage in crosstalk with extraverted immune cells to modulate their function (23). An increasing number of studies have identified the critical immunological function of pericytes in the CNS, but more investigations are needed to better understand the role of pericytes in BBB structure and function.

### Microglia

#### The Spatiotemporal and Phenotypic Dynamic of Microglia After AIS

The number of microglia in the infarct core decreased immediately after stroke. In addition, the number of microglia in the penumbra increased within hours after AIS and peaked at 48 - 72 hours, but microglia persisted in this region for several weeks (128). Microglia in the peri-infarct area were activated within minutes by DAMP, and these activated microglia in the penumbra became amoeboid- or round-like in morphology 12~24 hours after tMCAO (129). Proliferated microglia in the penumbra began migrating to the infarct core 1 day after stroke onset, which was mediated by the annexin-1/casein kinase II pathway (130).

Following morphological changes of microglia, the progression of intracellular signaling pathways and functions are differentiated between M1 pro-inflammatory microglia versus M2 anti-inflammatory microglia. Resident M2 microglia shifted to the M1 phenotype in the peri-infarct region in the early phase (within 48 hours) of ischemic stroke (131). High mobility group box-1 (HMGB1) proteins, heat shock proteins, and purines activated microglia by binding to TLR4 and purinergic receptor P2Y12. Activated microglia primed the nuclear transcription factor-kappa B (NF-kB) for translocation to the nucleus and to formation of the NLRP3 inflammasome, resulting in elevated levels of IL-1β (132). The triggering receptor expressed on the surface of myeloid cells 1 became elevated on the surface of microglia during ischemic stroke, activating CARD9/NF-κB, and NLRP3/caspase-1 proinflammatory pathway (133). M1-like microglia are characterized by the activation of inducible nitric oxide synthase (iNOS) and NF-кB, which produce NO, pro-inflammatory cytokines, and chemokines. The polarization of M2 microglia commenced several days after ischemic stroke and was induced by IL-4, IL-10, vascular endothelial-derived growth factor (VEGF), and GSF secreted from neurons (134). M2 type microglia acquire a phagocytic phenotype, which is indistinguishable from the phenotype of infiltrating macrophages due to the similarity in morphology and lack of specific markers between the two types of immune cells. The majority of macrophages present in the ischemic hemisphere 4-7 days following ischemic stroke derived from microglia (135). Recently, the comparative analysis of microglia-specific and monocytes-derived macrophage-specific transcripts revealed that microglia were more likely skewed toward transition to the M1 harmful phenotype compared to infiltrating macrophages in response to stroke (136).

#### Microglia Play a Dual Role in BBB Disruption in AIS

Microglia actively participate in the degradation of the BBB after ischemia (**Figure 2**). Following ischemic stroke, activated microglia in the penumbra engulfed blood vessels by expanding cellular protrusions toward vessels, enabling the extravasation of blood-borne macrophages. In addition, perivascular microglia engulfed ECs, leading to endothelial dysfunction and BBB disintegration (137).

M1 microglia enabled BBB disruption through the production and secretion of pro-inflammatory factors (IL-1α, IL-1β, IL-6, TNF-α, IFN-γ, CCL2), MMP9, VEGF, and ROS. IL-1α and IL-1β mediate their biological functions through binding to IL-1R1 expressed on nearly every cell in the brain. The expression of both IL-1α and IL-1β was dramatically elevated in the ischemic hemisphere 6-24 hours after stroke onset (138). Depletion of IL-1α or IL-1β attenuated BBB disruption and decreased infarct size in experimental stroke. IL-1α is a vital mediator of the activation of ECs to induce CXCL1 and IL-6 expression, and it induced AQP4 expression in astrocytes in ischemic tissue, which engendered both the deterioration of BBB and brain edema (139). Meanwhile, IL-1β increased the degradation and relocation of occluding and ZO-1 in ECs (140), and activated astrocytes with the over expressed GFAP by phosphorylating NF-кB p65. The astrocytes then upregulated VEGF expression after IL-1β stimulation (141). IL-1β also caused an increased secretion of CCL2, CCL20, and CXCL2, and it downregulated the expression of Sonic hedgehog, an important signal in maintaining BBB integrity in astrocytes (142).

Microglia-derived TNF-α induced endothelial necroptosis and the downregulation of occludin through binding to TNF receptor 1 (143). Meanwhile, TNF-α induced MMP9 expression but decreased the expression of collagen IV in ECs, both of which significantly increased BBB permeability *in vitro* (144). IFN-γ damaged the integrity of BBB by promoting the relocation of ZO-1 and VE-cadherin to cytoplasm in ECs (145). IL-1β, TNF-α, IFN-γ, and IL-6 increased the endothelial expression of ICAM-1 and VCAM-1, facilitating peripheral immune cell infiltration (145). Therefore, inhibition of microglial activation or blocking detrimental cytokines post-stroke may attenuate primary and secondary mechanism of BBB breakdown.

Like hematogenous macrophages, microglia develop an alternatively protective phenotype in the later phase of AIS. M2 microglia promoted inflammation resolution through the secretion of IL-4, IL-10, and TGF-β and the engulfment of immune cells, indirectly protecting against inflammation-induced BBB disruption. IL-4 and IL-10 restricted the expression of IFNγ, TNFα, and IL1β while elevating the levels of anti-inflammatory factors in the ischemic brain by inhibiting the activation of NF-κB (146). TGF-β decreased the levels of TNFα and MCP-1 through the ALK5-p-Smad2/3 signaling pathway (147). IL-10 decreased the expression of ICAM-1 and VCAM-1 in ECs, limiting the infiltration of immune cell into the brain (148). Microglia also secreted zinc finger E-box binding homeobox 1, which downregulated the expression of CXCL1 in astrocytes, thereby attenuating the infiltration of infiltration (149).

Microglia directly protect the BBB from damage through secretion of IL-10 and TGF-β in ischemic stroke. IL-10 protected ECs against ischemic injury by increasing the expression of γ‐glutamylcysteine synthase, a key enzyme in the synthesis of GSH and therefore, a critical player in maintaining the antioxidative status of cells (146). IL-10 also attenuated EC apoptosis by downregulating caspase-3 in the STAT3 pathway (150). Moreover, IL-10 limited the expression and activity of MMPs and enhanced the activity of tissue inhibitors of metalloproteinase (151). Meanwhile, TGF-β significantly prevented BBB leakage and HT by preventing the activity of MMPs and the degradation of ECM (152). Thus, IL-10 and TGF-β contribute to maintaining BBB structure and function in ischemic stroke.

Interestingly, IL-1 and TNF-α may also be participated in long-term BBB repair and angiogenesis in the chronic phase of AIS. IL-1 upregulated the expression of pentraxin-3 in the ischemic brain to promote glial scar formation and ECM production, maintainin BBB integrity and contributing to the resolution of brain edema (153). IL-1α promoted the proliferation and migration of ECs as well as the formation of tube-like structure (154). Microglia-derived TNF-α upregulated the expression of ephrin-A3 and ephrin-A4 in ECs to facilitate angiogenesis *in vitro* (155). In addition, TGF-β/ALK5 signaling attenuated infarction injury by promoting angiogenesis (156). Therefore, promoting the polarization of M2 microglia in the ischemic brain may accelerate the resolution of inflammation, BBB repair, and functional recovery.

### Role of Astrocytes in BBB Disruption in AIS

Emerging studies have suggested that astrocytes are not merely bystanders in immune responses after ischemic stroke. The activation of astrocytes during AIS, which is identified by GFAP positive immunostaining, is widespread and long-lasting. DAMP, including cytochrome c and ATP, activated NF-кB as well as induced the formation of the NLRP3 inflammasome and the release of IL-1β from astrocytes (157). A1 astrocytes were induced by pro-inflammatory factors, such as IL-1α and TNF-α, and the polarization of A1 astrocytes was characterized by the expression of iNOS, which could be modulated by LCN-2 in an autocrine manner (158). Microglia-derived anti-inflammatory factors transformed astrocytes into the protective A2 phenotype by downregulating the expression of P2Y1R in a brain trauma model (159). This indicated that A2 astrocytes might also be induced in the subacute phase of AIS. However, the spatiotemporal dynamic and the mechanism underlying the activation and transformation of astrocytes under acute ischemic injury have not been clarified, so more investigations are required in the future.

The terminology of ischemia-mediated two different phenotypes of A1 and A2 astrocytes parallel the terminology of M1 and M2 microglia in the CNS injury. Thus, similar to microglia, reactive astrocytes play dual roles in BBB breakdown after ischemic stroke.

On the one hand, astrocytes accelerate BBB disruption by amplifying inflammation injury and the secretion of soluble factors (**Figure 2**). Transcriptome analysis in reactive astrocytes found that genes involved in inflammation, leukocyte transendothelial migration, and JAK/STAT3 signaling were upregulated (160). Astrocytes directly exerted deleterious effects on the integrity of BBB by increasing the expression of VEGF, cytokines (IL-1β, IL-6, TNF-α, and IL-15), chemokines (CCL2 and CCL5), ROS, MMP, and LCN-2. Astrocyte-derived VEGF decreased the expression of TJs in ECs, which aggravated the BBB damage, infarction progression, and neurological deficits (161). Polymerase δ-interacting protein 2 was also upregulated in astrocytes following a stroke, which caused an increases in the extravasation of Evans blue by inducing the expression of TNF-α, IL-6, MCP-1, VEGF, and MMP (162).

Astrocytes also produce soluble factors to activate microglia and recruit peripheral immune cells, which indirectly potentiate the inflammation-induced BBB disruption. Lysophosphatidylcholine was found to increase CCL2 and CCR2 expression in microglia through G protein-coupled receptor 132 and P2X7R (163). Astrocyte-derived chemokines influenced microglia polarization in a paracrine manner through diverse chemokine receptors expressed on the microglia in AIS. In addition, astrocyte-derived VEGF aggregated the infiltration of peripheral immune cells by increasing the expression of ICAM-1 and VCAM-1 in ECs (161).

In contrast, astrocytes promote BBB repair by enabling the resolution of inflammation. A2 astrocytes are capable of secreting IL-2, IL-10, and TGF-β, leading to accelerated inflammation resolution. Pentraxin 3, released by astrocytes, attenuated IgG staining in ischemic cerebral tissue by inhibiting VEGF (164). Astrocytic insulin-like growth factor-1 (IGF-1) protected post-stroke BBB integrity and neurological function by shifting immune cells toward an anti-inflammatory profile in the ischemic environment (165). Astrocytes restricted microglia overactivation by upregulating the expression of CX3CR1 and IL-4Ra on the surface of microglia through TGF-β (166). However, TGF-β overexpression in astrocytes led to mural cell degeneration and dropout long-term (167).

Recent studies revealed that A2-specific transcripts were dominant over A1-specific transcripts 3 days following occlusion. Reactive A2 astrocytes expressed genes associated with scar formation and the regulation of ECM integrity, known as astrogliosis. Astrogliosis restricted the migration of infiltrating immune cells and limited the immune reaction within the infarct region (160). Surprisingly, growing evidence has shown that A2 astrocytes acquire the ability to engulf other cells under pathological situations. Reactive astrocytes in the penumbra were found to engulf and degrade cellular debris to assist with the resolution of inflammation *via* the ATP binding cassette A1 (ABCA1) after stroke (168). Depletion of ABCA1 in astrocytes profoundly increased BBB permeability and white matter lesions after ischemic stroke (169). Astrocytes exert such phagocytic activities in a delayed and prolonged manner. The phagocytotic activity of astrocytes typically starts 3 days post-ischemic stroke, peaks at 7 days, and persists for as long as 14 days.

### Role of Pericytes in BBB Disruption in AIS

Recently, a significant urge in attention has been paid to investigating the immunological role of pericytes in BBB disruption in ischemic stroke. In one investigation, TLRs were found to be constitutively expressed on the surface of pericytes, indicating that ischemia-induced DAMP might trigger NF-кB nuclear translocation and the upregulation of pro-inflammatory factors in pericytes (170). The sphingosine monophosphate receptor 2 was found to be involved in the NF-кB activation in pericytes during ischemic stroke by decreasing microRNA-149-5p levels, which was associated with increased BBB permeability (171). The activation of pericytes activation caused an increase in the expression of STAT3 binding genes as early as 2 hours after OGD (172). The immunological function of pericytes can also be modulated. For example, pericytes were capable of increasing the expression and secretion of cytokines (IL-6 and IL-8), chemokines (CX3CL1 and MCP-1), and adhesion molecules (VCAM-1) in response to TNF-a, IL-1β, IFN-γ, or TGF-β stimulation *in vitro* and in an inflammatory environment *in vivo* (173–175). In ischemic stroke, pericytes acquired a CD11b-positive inflammatory phenotype and upregulated the expression of the pro-inflammatory cytokine due to astrocyte-derived sema4D binding to plexinB1 receptor on the surface of pericytes, which contributed to increased BBB permeability after ischemic stroke (176). In addition, IL-1β-induced expression of MCP-1, IL-8, and ICAM-1 in pericytes was inhibited by the activation of transcription factor CCAAT/enhancer binding protein delta (CEBPD) (177). Thus, pericytes may adopt a biphasic phenotype during ischemic stroke, but this remains much to be explored.

Functioning as potential immune cells, pericytes mitigate BBB disruption in ischemic insult through the secretion of MMP, pro-inflammatory mediators, ROS, and laminin 5a. in an *in vitro* coculture of ECs and pericytes, the TEER value was significantly reduced under OGD conditions (178). In areas where pericytes adjoined ECs, a three-fold higher frequency of IgG leakage was observed in the photothrombotic occlusion model compared to areas without pericytes adjoined ECs. This rapidly increased BBB permeability might have been mediated by the activation of MMPs in pericytes (179). Pericytes strongly overexpressed inflammation-associated molecules, chemokines (CCL2 and CXCL10), and cytokines (IL-6), contributing to a worse outcome. The expression of pro-inflammatory molecules in pericytes is regulated by the transcription factor RBPJ (180). NADPH oxidase 4, an enzyme involved in the production of ROS, was overexpressed in pericytes in ischemic stroke, potentiating the degradation of TJs by increasing NF-кB and MMP activation (181). Laminin 5a in pericytes was detrimental to BBB disruption by amplifying uncontrolled infiltration of inflammatory cell in AIS (182). However, pericytes also improved BBB integrity by producing TGF-β (183).

Interestingly, pericytes, which feature Fc receptors, M2-marker (ED2), and scavenger receptors, behave like macrophages (184). The accumulation of lysosome-like granules in pericytes was observed in the human heart and animal brain, indicating that the pericytes were capable of phagocytizing other cells during AIS (185, 186). In addition, pericytes manifested a microglial-like phenotype that was abundant in activated-microglia-specific mRNA in ischemic stroke (187, 188). The phagocytic function of pericytes may promote inflammation resolution and BBB repair during ischemic stroke. However, whether pericytes are capable of demonstrating the microglia phenotype remains controversial (189). Nerve/glia antigen 2 (NG2) is a single membrane-spanning proteoglycan expressed by pericytes but not microglia in the healthy brain. Interestingly, NG2 immuno-positive microglia were detected in experimental stroke, which may support the possibility of pericytes differentiating into microglia. However, using three knock-in mouse lines, Huang et al. identified that activated microglia were expressed NG2 in an ischemic-dependent manner, indicating that pericytes could not differentiate into microglia in ischemic stroke.

## The Crosstalk Between Peripheral Immune Cells and Cerebral Immune Cells

Immune cells play an important role in BBB disruption during ischemic stroke. Moreover, the crosstalk between cerebral immune cells (CICs) and peripheral immune cells (PICs) forms a delicate and sophisticated network, the disturbance of which may indirectly influence BBB integrity during ischemic stroke.

CICs regulate the recruitment, extravasation, and function of PICs. For example, microglia, astrocytes, and pericytes upregulate and release diverse chemokines to motivate neutrophils, monocytes, and lymphocytes to the injured region after ischemic insult. Astrocytes attracted neutrophils in ischemic stroke by increasing the secretion of CXCL1 in response to the synergistic effects of TNF-α and IL-17A (114). CICs also secreted IL-1β and VEGF to stimulate the expression of VCAM-1 and ICAM-1 in the endothelium, which facilitates the attachment and infiltration of PICs. Owning to the unique location adjacent to microvascular ECs, astrocytes, and pericytes directly guide the extravasation of PICs. Both the chemoattractant MIF and ICAM-1 were upregulated in pericytes, which instructed the leukocytes infiltration pattern (190). Upon the interaction of pericytes with neutrophils, the cytoskeleton of pericytes became relaxed by inhibiting the RhoA/ROCK signaling pathway, which resulted in an enlargement of gaps between pericytes and low expression regions (LERs) of matrix proteins, thereby facilitating leukocyte extravasation (23, 191). Astrocytes provided the route guidance to infiltrated monocytes and macrophages through the interaction of fractalkine and CX3CR1 (192). After recognizing the invasion of neutrophil, microglia were observed to trap, contact, uptake, and finally engulf infiltrated neutrophils, diminishing the accumulation of neutrophils (193).

CICs also modulate the pro- or anti-inflammatory status of infiltrated PICs after ischemic stroke. M1 type microglia accelerate the polarization of Th1 cells by the secretion of IL-12 and TNF-α in the early stages of stroke. Meanwhile, microglia strongly induced the overexpression of HIF-1a/Sirtuin2 in Tregs through cell-to-cell contact, which inhibited the immunosuppressive function of Tregs (194). IL-15 derived from microglia and astrocytes increased the levels and activation of CD8^+^T cell and NK cell to potentiate BBB disruption in AIS (195). Astrocytes also polarized CD4^+^T cells into Th1 cells *in vitro* (196). Primary astrocytes were capable of maintaining FOXP3 expression in Tregs through IL-2/STAT5 signaling (197). Pericytes significantly inhibited the proliferation in and the secretion of pro-inflammatory factors from active T cells in the retina (198). Therefore, although there was no direct evidence in experimental stroke, astrocytes and pericytes may influence peripheral immune cells in ischemic conditions.

In turn, infiltrating PICs influence the polarization of CICs in ischemic stroke. Hematogenous monocytes and macrophages potentiated the polarization and process extension of astrocytes, promoting the BBB re-establishment (199). Microphage-derived exosomes polarized the microglia from the M1 pro-inflammatory phenotype to the M2 anti-inflammatory phenotype (200). Bone marrow-derived macrophages produced amphiregulin under ATP stimulation to change the status of pericytes by inducing TGF-β activation in acute lung injury, corroborating that the interactions between macrophages and pericytes could contribute to the BBB restoration in ischemic stroke (201). T lymphocyte subsets mainly exerted immunoregulatory effects in the ischemic brain. For instance, the Th1 subpopulation promoted M1 polarization of microglia through secretion of pro-inflammatory factors, such as IFNγ, which accelerated the amplification of inflammation in ischemic stroke. DNT increased the percentage of M1 pro-inflammatory microglia in the ischemic brain through the FasL/PTPN2/TNF-α pathway (202). Th2 cell-derived IL-4 inhibited the HMGB-1-induced expression of NF-κB and the formation of NLRP3 in astrocytes through STAT6/PPARγ singling (203). Th2 cells also potentiated the M2 polarization of microglia and the phagocytic capability of microglia in ischemic stroke through secreting IL-4 (204). Tregs inhibited the activation of microglia but promote the M2 polarization of microglia through recreation of IL-10 (197).

Given the complicated relationship between PICs and CICs, therapies targeting only one type of immune cell may prove deleterious or offset the benefit from another type of immune cell, resulting in an unsatisfied stroke prognosis.

## Immunotherapies Targeting BBB Disruption During Ischemic Stroke

### Therapies Targeting Neutrophils

#### Preclinical Attempts to Target Neutrophils for Treating AIS

Experimental investigations into targeting neutrophils for treating ischemic stroke have made tremendous breakthroughs in the past few decades. Modulating crucial neutrophil courses, including recruitment, adhesion, transmigration, and polarization, can significantly reverse severe BBB disruption and worse infarct damage (**Table 1**).

**Table 1:** Preclinical attempts to target neutrophils for treating AIS.

Mechanism	Drug	Molecular targets	Outcomes	Reference
Infiltration inhibition	reparixin	CXCR1 CXCR2	Reduced infarct volume; improved functional outcome	(205)
	evasin-3	CXCL1 CXCL2	No improvement in BBB leakage, infarct volume, or functional outcome	(206)
	Anti-CKLF1 antibody	CKLF1	Decreased BBB permeability	(207)
Adhesion interferes	Anti-ICAM-1 antibody/antisense oligonucleotides	ICAM-1	Decreased infarct volume and neurological deficit	(208)
	Anti-MAC-1 antibody (Hu23F2G)	Mac-1	Reduced ischemic injury	(209)
	Anti-E-selectin antibody	E-selectin	Decreased infarct volume and neurological deficit	(210)
	Anti-P-selectin antibody	P-selectin	Decreased BBB leakage, infarct volume, and neurological deficit	(211)
	anfibatide	GPIbα		(43)
	JAM-Ap	JAM-A		(212)
Deleterious factor neutralization	KYC	MPO		(213)
	Anti-lipocalin-2 antibody	lipocalin-2		(65)
	melatonin	MMP9		(214)
	S-oxiracetam			(215)
	alpha-1 antitrypsin	elastase	Decreased infarct volume and neurological deficit	(216)
Polarization regulation	All trans-retinoic acid	STAT1		(217)
	bexarotene	RXR/PPARγ	Decreased BBB leakage and infarct volume, improved neurological outcome	(218)
	rosiglitazone	PPARγ		(73)

CXCR1, C-X-C chemokine receptor 1; CXCR2, C-X-C chemokine receptor 2; CXCL1 CXC chemokines ligand 1; CXCL2, CXC chemokines ligand 1; BBB, blood-brain barrier; CKLF1, chemokine-like factor 1; G31P, ELR-CXC, CXC chemokines bearing the glutamic acid-leucine-arginine; ICAM-1, intercellular adhesion molecule-1; MAC-1, macrophage-1 antigen; GPIbα, glycoprotein Ib alpha; JAM-Ap, JAM-A antagonist peptide; JAM-A, Junctional adhesion molecule-A; KYC, N-acetyllysyltyrosylcysteine amide; MPO, myeloperoxidase; STAT1, signal transducer and activator of transcription; RXR/PPARγ, retinoid X receptors/peroxisome proliferator-activated receptor gamma.

Targeting neutrophil activation and recruitment may significantly reduce the infiltration of neutrophils into the ischemic hemisphere, but whether the treatment can improve BBB integrity and the functional outcome remains controversial. One study showed that CKLF1 inhibition alleviated MMP9 activity, ZO-1, and occludin disruption, as well as EB leakage after stroke (207). Reparixin, an inhibitor of CXCR1 and CXCR2, significantly decreased neutrophil extravasation and infarct volume and improved functional outcomes following ischemia-reperfusion (205). However, inhibiting CXCR2 alone did not attenuate infarction and motor impairment progression (60). Neutralizing the bioactivity of CXCL1 and CXCL2 by evasin-3 decreased neutrophil infiltration but did not reduce infarct volume, neurological deficit, or BBB permeability (206).

Recently, a novel strategy to redirect neutrophils from the ischemic brain to the periphery by peripherally implanting a CXCL1-soaked sponge led to improved neurological performance and infarction by reducing neutrophil-induced inflammation (219). Platelet-mimetic nanoparticles (PTNPs) were co-loaded with superparamagnetic iron oxide (SPIO), a T2 contrast agent. Piceatannol is a selective tyrosine kinase inhibitor. Like the CXCL1-soaked sponge, piceatannol, a selective tyrosine kinase inhibitor, can successfully recognize, intervene, monitor, and detach neutrophils into circulation, limiting infiltration of neutrophils in ischemia (220). Small extracellular vesicles derived from mesenchymal stromal cells protected against BBB disruption, ischemic injury, and neurological dysfunction, specifically by modulating the entry and accumulation of neutrophils (221).

Interfering with neutrophil rolling and adhesion by anti ICAM-1 (208) Mac-1 (209, 222), P-selectin/E-selectin (210, 211) antibody were effective in limiting the accumulation of neutrophils, BBB leakage, and ischemic injury in animals. The blocking of platelet glycoprotein receptor Ib decreased ischemic-induced BBB hyperpermeability through inactivation and downregulation of Mac-1 and P-selectin (43). In addition, inhibiting neutrophil-endothelia interactions with the JAM-A antagonist peptide reduced the expression of inflammatory mediators, BBB leakage, infarct size, and functional deficit (212).

Ameliorating the function of deleterious effectors derived from neutrophils protected the ischemic hemisphere from brain edema, infarction, and neurological deterioration. Blood substitution therapy profoundly improved stroke outcomes through the robust reduction of neutrophil, cytokine storm, and MMP-9 production. The benefits of blood substitution were diminished by the supplementation of MMP9 to the blood (223). Supplementing high-density lipoproteins or alpha-1 antitrypsin (an elastase inhibitor) inhibited neutrophil degranulation and the proteolysis of ECM (224). The diminishment of LCN-2 by antibodies significantly reduced brain edema, BBB permeability, and functional outcome after stroke injury (65). Inhibition of MPO by *N*-acetyl lysyltyrosylcysteine amide significantly decreases IgG extravasation and neurological severity score in stroke (213). Administration of either melatonin or S-oxiracetam maintained BBB integrity, which was partly attributed to inhibiting the activity of deleterious factors from neutrophils in the MCAO model (214, 215).

Lastly, targeting the polarization of neutrophils offers a promising target to attenuate ischemic damage to BBB. All-*trans*-retinoic acid prevented BBB disruption, infarction, and behavior deficits by skewing neutrophils toward the N2 phenotype by suppressing STAT1 (217). Retinoid X receptor (RXR)/PPARγ agonists, including bexarotene and rosiglitazone, elevated the number of Ym1 positive neutrophils by inhibiting NF-κB transactivation, which was also associated with the attenuation of BBB leakage, infarction enlargement, and neurological deficits (73, 218).

#### Clinical Trials Targeting Neutrophils in AIS

Inspired by the positive results from inhibiting adhesion molecules in animals, targeting neutrophil adhesion in patients suffering ischemic stroke has been widely evaluated in clinical trials (**Table 2**). Unfortunately, they all failed. Enlimomab (anti-ICAM-1 antibody)-treated patients had worse functional outcomes and more adverse events than placebo-treated patients in AIS (235). Administration of UK-279,276, a Mac-1 inhibitor, did not improve recovery from AIS (236). In the LeukArrest study, clinical trials of Hu23F23F2G, which targeted Mac-1 and LFA-1, terminated early without final reports (237). Clinical trial to evaluate the safety and effectiveness of E-Selectin tolerance induction in patients who have had a transient ischemic attack or stroke was suspended without available results (238).

**Table 2:** Randomized controlled trials targeting immune cells in human ischemic stroke.

Treatment	Mechanism	Author	Year	Study place	Sample Size	Intervention			Outcome				Reference
						Time window, duration	Drug dose	Administration route	mRS/ΔSSS/mBI at day 90	Mortality rate	Infarct volume at day 5/NIHSS score at 1 week	Adverse event	
Anti-ICAM-1 (Enlimomab)	Inhibiting leukocytes infiltration	Enlimomab Acute Stroke Trial Investigators	2001	USA Europe	Enlimomab (n=317) placebo (n=308)	Within 6 hours after onset; 5 days	LB: 160mg; MB: 40mg	Intravenously	worse	Higher	-^*^	More infections and fever	(214)
Anti-Mac-1(UK-279,276)	Inhibiting leukocytes infiltration	Krams	2003	UK	966 patients randomly treated	Once within 6 hours after onset	1 of 15 doses (dose range, 10 to 120 mg)	Intravenously	Not significant	Similar	–	More headache	(215)
Anti-α4 integrin (natalizumab)	Inhibiting lymphocyte infiltration	Elkins	2017	USA Europe	Natalizumab (n=79) Placebo (n=82)	Once up to 9 h after onset	300 mg	Intravenously	More good outcome	–	Similar	Similar	(225)
Anti-α4 integrin (natalizumab)	Inhibiting lymphocyte infiltration	Elkind	2020	USA Europe	300mg Natalizumab (n=91) 600mg Natalizumab (n=92) Placebo(n=94)	≤9 or >9 to ≤24 hours	300 or 600 mg	Intravenously	Similar	Similar	–	Similar	(226)
S1PR agonist (fingolimod)	Inhibiting lymphocyte infiltration	Fu	2014	China	Fingolimod (n=11) Control (n=11)	Within 72 hours after onset; 3 days	0.5mg	Orally	More good outcome	No	Less	No serious event	(227)
S1PR agonist (fingolimod)	Inhibiting lymphocyte infiltration	Zhu	2015	China	Fingolimod (n=25) Control (n=22)	Within 4.5 hours after onset; 3 days	0.5mg	Orally	More good outcome	–	Less	No serious event	(228)
S1PR agonist (fingolimod)	Inhibiting lymphocyte infiltration	Tian	2018	China	Fingolimod (n=23) Control (n=23)	4.5-6 hours after onset; 3 days	0.5mg	Orally	More good outcome	No death	Less	No serious event	(226)
Resveratrol	Regulating lymphocyte subsets	Chen	2016	China	Resveratrol (n=154) Placebo (n=158)	Within 4 hours after onset	2.5 mg/kg	Intravenously	Only 24 hours NIHSS score is evaluated, Resveratrol reduce NIHSS score at 24 hours				(229)
Atorvastatin	Regulating lymphocyte subsets	Muscari	2011	Italy	Atorvastatin (n=31) Placebo (n=31)	Within 24 hours after onset; 7 days	80mg	Orally	More good outcome	Similar	Similar	Similar	(230)
Rosuvastatin	Inhibiting microglia activation	Heo	2016	Korea	Rosuvastatin (n=155) Placebo (n=159)	Within 66 hours after onset; 14 days	20 mg	Orally	–	Similar	Similar	Similar	(231)
Minocycline	Inhibiting immune cells activation	Westphal	2007	Israel	Minocycline (n=74) Placebo (n=77)	6-24 hours after stroke onset; 5 days	200 mg	Orally	More good outcome	Similar	–	Similar	(232)
IL-1 receptor antagonist (rhIL-1ra)	Inhibiting IL-1	Emsley	2005	UK	rhIL-1ra (n=17) Placebo (n=17)	Within 6 hours after onset; 3 days	LB: 100mg; MB: 2mg/kg/h	Intravenously	More good outcome in cortical infarcts patients	Similar	Less in cortical infarcts patients	Similar	(233)
IL-1 Receptor Antagonist (IL-1Ra)	Inhibiting IL-1	Smith	2018	UK	IL-1Ra (n=39) Placebo (n=41)	Within 5 hours after onset; 3 days	100 mg twice daily	subcutaneously	Similar	Similar	Similar	Similar	(234)

*not mentioned in the study.

AIS, acute ischemic stroke; mRS, modified ed Rankin Scale; ΔSSS, the Scandinavian Stroke Scale; mBI, modified Barthel Index; NIHSS, the National Institute of Health Stroke Scale; ICAM-1, intercellular adhesion molecule-1; USA, The United States of America; LB, loading bonus; MB: maintenance bolus; RCT, Randomized Controlled Trial; MAC-1, macrophage-1 antigen; UK, The United Kingdom; S1PR, sphingosine 1-phosphate receptor; rhIL-1ra, recombinant interleukin-1 receptor antagonist.

Some of these potential drugs discussed were safe and efficacious against other diseased, but they require more clinical trials for determining their efficacy in treating AIS. For instance, the safety and efficacy of reparixin were demonstrated in treating breast cancer recently (239). Melatonin supplementation reduced myocardial ischemic-reperfusion injury in a dose-dependent manner (240). In addition, all *trans*-retinoic acid might be an alternative therapy for leukemia (241).

### Therapies Targeting Monocytes

#### Preclinical Attempts to Target Monocytes for Treating AIS

Skewing monocyte-derived macrophages toward the alternatively activated M2 phenotype can be a potential and effective target for AIS treatment (**Table 3**). Administration of P2X4R antagonist 5-BDBD was found to significantly reduce BBB leakage, infarct size, and neurological deficit (242). Propane-2-sulfonic acid octadec-9-enyl-amide (243), 1, 25-dihydroxyvitamin D3 (244), rosiglitazone (245), and recombinant human fibroblast growth factor 21 (270) all upregulated the expression of Arg-1,Ym1, and CD206 and downregulated the expression of iNOS in microglia and macrophages by activating PPARγ. These PPARγ agonists profoundly maintained the TJs and BBB integrity, improved infarction injury and neurological score. The activation of GPR35 by pamoic acid increased the number of alternative monocytes and macrophages in the ischemic hemisphere and reduced the infarct volume and functional disorder through the protein kinase B (Akt)/p38 MAPK signaling pathway (247). Other modulators, such as DHA (248), 12-(3-adamantan-1-yl-ureido)-dodecanoic acid (250), miR-669c (251), significantly elevated the expression of Arg1 and reduced infarct volume and neurological disorder caused by cerebral ischemia.

**Table 3:** Preclinical attempts to target monocyte and microglia for treating AIS.

Target cell	Mechanism	Drug	Molecular targets	Outcomes	Reference
monocytes	Polarization regulation	5-BDBD	P2X4R	Reduced BBB leakage, infarct size neurological deficit	(242)
		N15	PPARα/γ		(243)
		1, 25-D 3	PPARγ		(244)
		rosiglitazone		Reduced BBB leakage and hemorrhagic transformation	(245)
		rhFGF21		Reduced infarct size and neurological deficit	(246)
		pamoic acid	GPR35		(247)
		DHA	?		(248, 249)
		AUDA	sEH		(250)
		miR-669c	MyD88		(251)
Microglia	Regulation of Activation and Polarization	ligustilide	TLR4		(252)
		miR-1906			(253)
		TAK-242			(254)
		eritoran		Reduced BBB disruption and infarct volume; improve functional outcome	(255)
		MTS510			(256)
		Ticagrelor	P2Y12R		(257)
		adjudin	NF-kB		(258)
		Statin	HMG-CoA reductase		(259–261)
		Minocycline	NLRP3		(262–265)
		Metformin	AMPK		(266–268)
	Cytokine inhibition	IL-1Ra	IL-1		(231)
		Canakinumab	IL-1β		(269)
		Infliximab	TNF-α		(143)

P2X4R, purinergic P2Y4 receptor; BBB, blood brain barrier; N15, Propane-2-sulfonic acid octadec-9-enyl-amide; PPARα/γ, peroxisome proliferator-activated receptor alpha/gamma; 1, 25-D 3, 1, 25-dihydroxyvitamin D3; rhFGF21, recombinant human fibroblast growth factor 21; NF-κB, nuclear factor kappa B; GPR35, G protein Coupled Receptor 35; Akt/p38 protein kinase B/p38 MAPK, mitogen-activated protein kinases; DHA, docosahexaenoic acid; AUDA, 12-(3-adamantan-1-yl-ureido)-dodecanoic acid; sEH, soluble epoxide hydrolase; MyD88, myeloid differentiation primary response gene 88; TLR4, Toll-like Receptor 4; P2Y12R, purinergic P2Y12 receptor;NLRP3, NOD-like receptor family pyrin domain containing 3; AMPK, AMP-activated protein kinase; IL-1Ra, interleukin-1 receptor antagonist; IL-1, interleukin-1; IL-1β, interleukin-1β; TNF-α, tumor necrosis factor-α.

#### Clinical Trials Targeting Monocytes in AIS

There is no drug that directly targets monocytes in clinical ischemic stroke currently. Due to similar polarization mechanism and function with microglia, drugs that regulate microglia activation and polarization may also interfere monocytes status in AIS (see Part 5.4.2 *Clinical Trials Targeting Microglia in AIS*). Further studies are warranted for developing strategies to modulate monocytes in AIS.

### Therapies Targeting T Lymphocytes

#### Preclinical Attempts to Target T Lymphocytes for Treating AIS

The therapeutic options for AIS that targets T lymphocytes involve three pathways (**Table 4**). The first entails decreasing the infiltration of T lymphocytes.

**Table 4:** Preclinical attempts to target T lymphocytes for treating AIS.

Mechanism	Drug	Molecular targets	Outcomes	Reference
Infiltration inhibition	Anti-VLA-4 antibody	CD49d	Reduced infarct size and neurological deficit	(271)
	FTY720/Fingolimod	S1P R	Reduced hemorrhagic transformation	(272, 273)
Immune homeostasis regulation	JPI-289	PARP-1	Reduced BBB leakage, infarct size, and neurological deficit	(274)
	PJ34			(275)
	FR247304		Reduced infarct size, neurological deficit	(276)
	MP-124			(277)
	IL-33	ST2R		(278–280)
	vitamin D 3	vitamin D receptor		(281)
	atorvastatin	HMG-CoA reductase		(282)
	resveratrol	PPARγ		(283)
Gut microbiota modulation	resveratrol	Microbiota formation	Reduced BBB leakage, infarct size, neurological deficit	(284)
	fecal microbiota transplantation			(225, 285)
	NaB	HDAC		(227)
	VPA			(228)

VLA-4, very late antigen-4; CD49d, cluster of differentiation 49d; S1P R, sphingosine 1-phosphate receptor; BBB, blood brain barrier; PARP-1, Poly (ADP-ribose) (PAR) polymerase-1; IL-33, interleukin-33; ST2R, simultaneous translation on 2 rods; PPARγ, peroxisome proliferator-activated receptor gamma; NaB, Sodium butyrate; HDAC, histone deacetylase; VPA, valproic acid.

Blocking the infiltration of T lymphocytes by inhibiting CD49d (integrin α4) reduced the overall infiltrated T cell count, while the terminal stroke outcome is controversial (271, 286). FTY720 (Fingolimod), a sphingosine-1-phosphate (S1P) receptor agonist, is a strong candidate for AIS treatment because it blocks the motivation of lymphocytes from lymph nodes to the bloodstream (287). Fingolimod administration improved the BBB integrity, infarct injury, and functional outcome (272, 273).

The second pathway entails manipulating the imbalance between pro-inflammatory subsets and anti-inflammatory subsets. Changing the T cell subpopulation disorder rescued BBB hyperpermeability after ischemic stroke. Poly (ADP-ribose) polymerase-1 (PARP-1) is an abundant nuclear protein involved in DNA repair. Inhibitors of PARP-1, namely JPI-289, FR247304, and MP-124, significantly maintained the TJs and reduced the MMP9 levels, brain swelling, HT occurrence, infarction size, and functional deficiency in stroke (274, 276, 277). The post-stroke suppression of acetyl coenzyme A carboxylase 1(ACC1), a critical metabolic enzyme involved in fatty acid synthesis, maintained the ratios of Treg to Th17, which reduced the inflammation and ischemic lesion (288). Injection of recombinant IL-33 into mice dramatically attenuated the deterioration of brain edema and neurological deficit by maintaining the levels and activities of Th2 cells and Tregs and dampening the counts and immune responses of Th1 and Th17 cells (278, 279). IL-33 induced TGF-β and IL-10 elevation *via* ligation to the ST2 receptor (279). Supplementing vitamin D 3, resveratrol, or atorvastatin prevented infarction and neurological dysfunction by shifting immune balance to enhanced Treg reaction and depressed Th17 and γδT response (281–283). The effect of atorvastatin on Treg expansion was concentration-dependent, as Treg immunosuppression was hindered at a high concentration of atorvastatin *in vitro* (289).

The third pathway involves regulating the composition of gut microbiota. Targeting the gut microbiota to protect the BBB and the brain against ischemic insult has demonstrated strong potential in animals. Resveratrol robustly attenuated the inflammation-induced degradation of BBB by regulating the intestinal flora (284).

The post stroke transplantation of beneficial fecal microbiota, abundant in butyric acid and short-chain fatty acids, namely *Clostridium butyricum, Bifidobacterium longum, Lactobacillus fermentum*, and *Faecalibacterium prausnitzii*, significantly alleviated post-stroke BBB disruption, infarct size, and neurological deficits in mice (225, 285). In addition, supplementing microbial metabolites, such as short-chain fatty acids (SCFAs), in ischemic stroke maintained the balance of pro- and anti-inflammatory subsets by inhibiting the activity of histone deacetylase (HDAC). Furthermore, HDAC inhibitors, including sodium butyrate and valproic acid, protected against stroke-induced BBB disruption and brain edema by inhibiting MMP9 production, TJ degradation, and NF-kB activation (227, 228).

#### Clinical Trials Targeting T Lymphocytes in AIS

Natalizumab is a monoclonal antibody targeting α4 integrin to block the adhesion and infiltration of lymphocytes. Natalizumab administration in a prior phase 2 trial (ACTION) failed to reduce infarct volume but improved functional outcome 90 days after AIS (290). However, the benefits on functional outcomes were not seen in ACTION II (226). Combining results of ACTION and ACTION II cannot support the evidence of the benefit of natalizumab for AIS (**Table 2**). FTY720 is a promising drug for improving stroke outcomes by its ability to decrease the infiltration of lymphocytes. Several pilot trials and randomized control trials have verified the safety and efficacy of FTY720 in patients with AIS as a single therapy that exceeded the therapeutic window. FTY720 treatment combined with alteplase thrombolysis also improved stroke outcomes by decreasing microvascular permeability (229, 291–293). The mechanisms underlying the efficacy of fingolimod in treating AIS are multifaced and are reviewed in (287).

Regulating the homeostasis of T lymphocyte subsets in patients might be a hopeful treatment for AIS. One study discovered that inhibiting PARP-1 increased the proportion of Treg and Treg-relevant transcription factors (i.e., FoxP3 and CTLA-4 mRNA) in the blood of ischemic patients (100). Meanwhile, PARP-1 inhibition reversed the disbalance between pro-inflammatory factor (IL-17, IFN-γ, and TNF-α), and anti-inflammatory factors (TGF-β, IL-4, and IL-10). The safety and tolerance of JPI-289, a PPAR-1 inhibitor, have been successfully demonstrated in healthy volunteers recently, which served as a foundation for further related clinical trials (294). Resveratrol administration significantly improved the functional outcome in stroke patients (230). However, oral supplementation with vitamin D 3 failed to decrease the risk of stroke occurrence and did not improve stroke outcomes in patients long-term (295, 296). Randomized placebo-controlled trials revealed that atorvastatin did not decrease the infarct volume or improve functional behavior in AIS patients (297, 298).

### Therapies Targeting Microglia

#### Preclinical Attempts to Target Microglia for Treating AIS

The discovery of therapies targeting microglia for the treatment of AIS is of great interest for inhibiting microglial activation and regulating microglia polarization (**Table 3**).

PRR ablation shielded microglia from activation after ischemic stroke. Blocking TLR4 with eritoran, MTS510, ligustilide, miR-1906, and TAK-242 reduced the number of activated microglia and the production of iNOS, ROS, and pro-inflammatory factors, thereby protecting the BBB against inflammatory injury in stroke (252–256). Ticagrelor, a reversible antagonist of P2Y12R, significantly attenuated BBB damage by decreasing the activation of microglial and lowering the level of IL-1β, CCL2/MCP-1, and iNOS (257). Teriflunomide reserved pericytes coverage and TJ expression by reducing microglial activation and downregulating cyclooxygenase-2 and IL-1β 3 days following tMCAO (299). Interfering with NF-kB signaling through supplementation with adjudin significantly inhibited the activation of microglia, overexpression of TNF-α, IL-1β and IL-6, and the disruption of ZO-1, occluding, and JAM-A. Adjudin decreased the IgG extravasation, infarct size, and functional disorder in experimental stroke (258). In addition, statin therapy significantly reduced BBB breakdown, brain swelling, and functional deficit by decreasing microglial activation (259, 300).

Suppressing the NLRP3 inflammasome promoted the M2 polarization of microglia. Minocycline, a tetracycline antibiotic that demonstrated anti-inflammatory effects in neurodegenerative diseases inhibited microglial activation and promoted microglia M2 polarization by suppressing the NLRP3 inflammasome (262). Minocycline increased the expression of IL-10, TGF-β, Ym-1, and Arg-1 but decreased the expression of IL-1β, IL-6 andTNF-a (263). Thus, minocycline administration significantly reduced BBB leakage, brain edema, infarct volume, and neurological dysfunction (264). Metformin, which is widely used in clinical for treating diabetes, activated adenosine 5′monophosphate-activated protein kinase (AMPK). Metformin not only attenuated post-stroke BBB damage but also promoted angiogenesis and recovery in a microglia M2 polarization-dependent manner (266–268). In addition, PPARγ agonist discussed preciously potentiated polarization of microglia to M2 phenotype.

Blocking the production of harmful cytokines during ischemic stroke may also be a promising therapeutic target for treating AIS. IL-1R antagonist (IL-1Ra) is a natural inhibitor of IL-1 signaling. IL-1Ra administration reduced inflammation-induced BBB disruption, infarct size, and neurological deficit in rats (231). Cell therapy based on IL-1Ra was also effective in treating ischemic stroke (301). Canakinumab, an inhibitor of IL-1β, significantly decreased brain edema, infarct volume, and functional deficit in a murine tMCAO model (269). Anti TNF-α (infliximab) therapy significantly enabled the preservation of BBB structure and behavioral performance in experimental stroke (143). However, further investigation is required to evaluate the efficacy and safety of biomimetics and antibodies against pro-inflammatory cytokines in treating AIS.

#### Clinical Trials Targeting Microglia in AIS

Regulating the activation and polarization of microglia in AIS patients has been identified as an effective strategy to improve the prognosis of AIS (**Table 2**). The use of statin in patients with AIS was found to be safe and well-tolerated. Patients receiving statins after ischemic stroke onset displayed reduced microemboli and HT (233, 234). Minocycline treatment in patients with AIS markedly improved the patients’ functional outcome after 3 months, which was based on mRS scores of 0-2 (302). Patients who were administered metformin before ischemic stroke showed significantly lower stroke severity, mRS score, and mortality compared to those who were not given metformin (303).

Inhibiting pro-inflammatory cytokines is also helpful for clinical intervention of ischemic stroke. Supplementing with IL-1Ra was determined to be safe and well-tolerated in ischemic stroke patients. Recombinant human IL-1ra (rhIL-1ra) administered intravenously in ischemic stroke patients displayed profoundly lower inflammatory biomarkers in their serum and better clinical outcomes 3 months after ischemic stroke (304). IL-1Ra administered subcutaneously was not associated with an improved functional outcome; however, it significantly reduced the presence of circulating inflammatory markers (305). Antibodies against IL-1β and TNF-α were not evaluated in ischemic stroke patients. CANTOS Trial demonstrated that canakinumab significantly reduced atherothrombotic cardiovascular events in 10,061 patients (306). Lastly, infliximab and its biosimilar were widely investigated in patients with autoimmune diseases (307).

### Therapies Targeting Astrocytes

#### Preclinical Attempts to Target Astrocytes for Treating AIS

Given the essential role of astrocytes, whether deleterious or beneficial, in BBB disruption during AIS, astrocytes can be potential therapeutic targets. Treatments targeting astrocytes involve decreasing astrocyte activation and inhibiting the detrimental factors from astrocytes (**Table 5**).

**Table 5:** Preclinical attempts to target astrocytes for treating AIS.

Mechanism	Drug	Molecular targets	Outcomes	Reference
Inhibiting Activation	Cottonseed oil	TLR	Decreased BBB disruption, infarction size, and alleviated functional disorder	(308)
	Ginkgoaceae			(309)
	Metformin		Reduced infarct volume and neurological deficit	(310)
	Ligustilide			(252)
	Z-Guggulsterone			(311)
	MicroRNA-1906			(253)
	Kaempferol glycosides			(312)
	Honokiol		Reduced BBB permeability	(313)
	IL-32a		Improved functional outcome	(314)
	Telmisartan	NLRP3	Reduced GFAP positive astrocytes	(315)
	Sinomenine		Alleviated cerebral infarction, BBB disruption and neurological deficit	(316)
	Adiponectin			(317)
	Afobazole	Sigma receptor	Reduced astrocytes activation	(318)
	PRE-084		Decreased infarct volume and neurological deficiency	(319)
	AS605240	PI3Kγ	Improved neurological function and reduced infarct size	(320)
	Oleoylethanolamide	PPARα	Improved motor function	(321)
Diminishing Detrimental factors	Memantine	MMP2/9	Reduced BBB leakage, infarct size and neurological defecit	(322)
	Exendin-4	GLP-1R		(323)
	IGF-1 supplement	IGF-1	Improved BBB integrity and sensory-motor performance	(324)

TLR, Toll like receptor; BBB, blood brain barrier; IL-32a, interleukin-32a; NLRP3, NOD-like receptor family pyrin domain containing 3; GFAP, glial fibrillary acidic protein; PI3Kγ, phosphoinositide 3-kinase gamma; PPARα, peroxisome proliferator-activated receptor alpha; MMP, matrix metalloproteinases; GLP-1R, the glucagon-like peptide-1 receptor; IGF-1, insulin-like growth factor-1.

Blocking the activation of astrocytes in ischemic stroke may prevent BBB disruption and promote proper neurological performance. Given the similarities in activation signaling between astrocytes and microglia, some drugs that function to inhibit immune cell activation are efficacious against both microglia and astrocytes. TLR-NF-kB signaling is the initial step in astrocyte activation. By inhibiting TLR and NF-kB expression, cottonseed oil (308), *Ginkgoaceae* (309), metformin (310), ligustilide (252), *Z*-guggulsterone (311), IL-32a (314), microRNA-1906 (253), honokiol (313), and kaempferol glycosides (312), all reduced the number of reactive astrocytes and the levels of IL-1β, IL-6, and TNF-α, which significantly attenuated BBB disruption, brain edema, and functional neurological performance. NLRP3 inflammasome is an important signaling mechanism involved in the activation of A1 astrocytes. Thus, many drugs, such as telmisartan (315), sinomenine (316), and adiponectin (317) alleviated A1 astrocytes-induced BBB disruption and neurological deficit by inhibiting NLRP3. In addition to afobazole and PRE-084, animals treated with the sigma receptor agonist showed elevated astrocyte numbers but decreased proportion of reactive astrocytes in the ischemic hemisphere, leading to a small infarct volume (318, 319). AS605240, a PI3Kγ inhibitor, significantly reduced the astrocyte activation and morphological changes as well as the expression of pro-inflammatory factors, resulting in improved stroke outcome (320). Oleoylethanolamide promoted the expression and nuclear transportation of PPARα in astrocytes to inhibit astrocyte activation and neurological deficit in the ischemic hemisphere (321).

Diminishing astrocytic detrimental factors also protected the BBB against inflammatory injury. Vagus nerve stimulation and memantine administration attenuated BBB leakage and infarct size by reducing astroglia MMP2/9 expression (322, 325). Exendin-4, a glucagon-like peptide-1 receptor agonist, decreased the HT by reducing the expression of astrocyte-derived MCP-1, CXCL1, VEGF, and MMP9 through the reduction of highly glycosylated CD147, an MMP inducer (323, 326) Supplementing with IGF-1 also improved BBB integrity and sensory-motor performance in a murine model of tMCAO (324).

#### Clinical Trials Targeting Astrocytes in AIS

Some drugs mentioned above have already been approved by the FDA or the Chinese government for other diseases. For example, telmisartan is widely used as an angiotensin-receptor blocker for the treatment of hypertension. Sinomenine is the main active ingredient in the plant *Sinomenium acutum* and has been used to treat rheumatic and arthritic diseases in China for over 1000 years (327). Afobazole, an anxiolytic drug, used by patients with generalized anxiety disorder. Memantine is an *N*-methyl-D-aspartate (NMDA) receptor antagonist approved for the treatment of Alzheimer’s disease. Exenatide is a synthetic product of the glucagon-like peptide-1 analogue exendin-4 and is clinically utilized for patients with type 2 diabetes.

Among these candidates, the safety and efficiency of telmisartan and exenatide in treating AIS have been evaluated through clinical trials. Exenatide behaved well in controlling post-stroke hyperglycemia without worsening neurological outcomes (328). Currently, the superiority of exenatide treatment compared to standard treatment is being evaluated in a phase 2 trial as a potential treatment for AIS (329). In the Prevention Regimen for Effectively Avoiding Second Strokes (PRoFESS) trial, telmisartan utilization did not alter the neurological deficiencies in patients with recurrent stroke (330). Considering these recent advancements, while drug repositioning or repurposing may be relatively inexpensive and reliable in AIS compared to the development of new, specific therapeutics, more investigations related to the discovery of new drugs for treating AIS are warranted.

### Therapies Targeting Pericytes

Potential immunological therapies targeting pericytes in ischemic stroke have not been explored deeply due to the uncomprehensive understanding of their role. From the information discussed above, it has been hypothesized that modulating the inflammatory status of pericytes against the secretion of detrimental factors and promoting the phagocytotic properties of pericytes may limit inflammation-induced BBB leakage to promote functional recovery in ischemic stroke.

## Discussion

Currently, only intravenous thrombolysis with rt-PA and MT are effective treatments for acute ischemic stroke (11). However, they are limited by only being efficacious in a narrow treatment window. Neuron-centric neuroprotective therapies for treating AIS have failed. Therefore, it is crucial to discover new therapeutic targets for ischemic stroke. BBB disruption often engenders a poor prognosis and mortality in patients with AIS. Because both cerebral and circulating immune cells play a complex and lasting role in BBB destruction, immunotherapies targeting BBB disruption can be adjusted accordingly at various time points during the treatment of AIS.

Peripheral immune cells are recruited and infiltrate into the brain after ischemic stroke. Neutrophils have long been considered a destructive effector in BBB disruption. Interestingly, the other protective classification of neutrophils was discovered recently. Monocytes have a dual role in BBB destruction after cerebral infarction through the ability to differentiate into M1 and M2 macrophages. Promoting the differentiation into M2 macrophages has become a hot spot and potential target for therapeutic exploration. T lymphocytes have a tendency to differentiate into pro-inflammatory subgroups after cerebral infarction, which damage BBB integrity. Thus, identifying the driver and regulator of T lymphocytes pool differentiation after cerebral infarction is advantageous to the discovery of novel and effective therapeutic targets.

Cerebral immune cells are activated rapidly after artery occlusion. Perivascular immune cells can directly influence BBB integrity in stroke. Similar to monocytes, microglia polarization has gained significant attention over the years and may be a promising target in treating ischemic stroke. Astrocytes are also innate immune cells and regulate the course of inflammation and BBB disruption through the secretion of diverse soluble molecules. Recently, researchers have discovered that the immunological effects of pericytes after AIS is beyond previous thoughts. However, it remains unclear how astrocytes and pericytes regulate BBB stability using the immune system.

There are two main bottlenecks that impede the clinical development of immunoregulatory therapies targeting BBB disruption. One is the mismatch between clinical stroke and experimental stroke. The efficacy of immunoregulatory treatments in animals (experimental stroke) was based on the tMCAO model (331). However, the majority of AIS patients underwent permanent ischemia without reperfusion. The other is the difficulty of knowing when to start and terminate immune intervention in ischemic stroke. Most immune cells do not have a single role during the course of AIS. Treatment with immunoregulatory drugs must be easy to stop to prevent further offsetting the benefit from an early stage of AIS. Moreover, circulating and cerebral immune cells form a delicate and sophisticated inflammatory network. Therapies targeting only one type of immune cell may prove deleterious or offset the benefit from another type of immune cell, resulting in an unsatisfied stroke prognosis. However, further exploration is needed to identify effective treatment measures.

Overall, targeting immune cells in the BBB disruption in AIS is a promising strategy to improve stroke prognosis and improve existing treatments. More investigations using animal models that better simulate human stroke are warranted. In addition, detailed mechanisms underlying the inflammatory mechanism of BBB disruption remain to be uncovered.

## Author Contributions

Y-mQ and C-lZ wrote the manuscript. Y-nL and BH conceived this article. A-qC and H-lW drew the chart. Y-fZ helped revise the manuscript and check the grammar. All authors contributed to the article and approved the submitted version.

## Funding

This work was supported by the National Natural Science Foundation of China (No. 82071336 to YNL), Natural Science Foundation of Hubei Province (No. 2020CF763 to YNL), National Key R&D Program of China (No. 2018YFC1312200), National Natural Science Foundation of China (No. 82090044 to BH, No. 81820108010 to BH, No. 81901212 to YFZ).

## Conflict of Interest

The authors declare that the research was conducted in the absence of any commercial or financial relationships that could be construed as a potential conflict of interest.
